# Consequences of mRNA Secondary Structure on Stability against both Hydrolysis and Aggregation: The Role of Electrostatic, π–π Stacking, and Thermal Effects

**DOI:** 10.1021/acsomega.5c10266

**Published:** 2026-01-08

**Authors:** Curtis W. Jarand, Zhiyou Deng, Mark L. Brader, Wayne F. Reed

**Affiliations:** 1 5783 Tulane University, New Orleans, Louisiana 70118, United States; 2 692728 Moderna, Inc., Cambridge, Massachusetts 02141-2025, United States

## Abstract

The seemingly unrelated massive aggregation of free mRNA under certain solution conditions and the well-known autohydrolysis of mRNA are actually both closely linked through its secondary and possibly tertiary structure (s/t). This hypothesis posits that s/t partially stabilizes mRNA against both autohydrolysis and massive aggregation. Destabilization of s/t via denaturant guanidine-HCl (Gd), or temperature, has profound effects on both aggregation rates and final degree of autohydrolysis. These denaturant effects occurred for a variety of mRNA, ranging from 700 to 3000 nucleotides but showed very different quantitative behavior among themselves, suggesting some of the methods presented here might help characterize mRNA stability and robustness. Light scattering monitoring during dialysis of mRNA against Gd revealed an “aggregation window”, over 0.5–3 M Gd, whereas dialyzing against a nondenaturing electrolyte (NaCl) showed semireversible monotonic increase of aggregation up to 4 M. Massive aggregation of mRNA in solutions with monovalent ions and in denaturing solutions has not been previously reported. A phenomenological model involving intermolecular electrostatic repulsion and attractions due chiefly to π–π stacking helps interpret the various phenomena.

## Graphical abstract

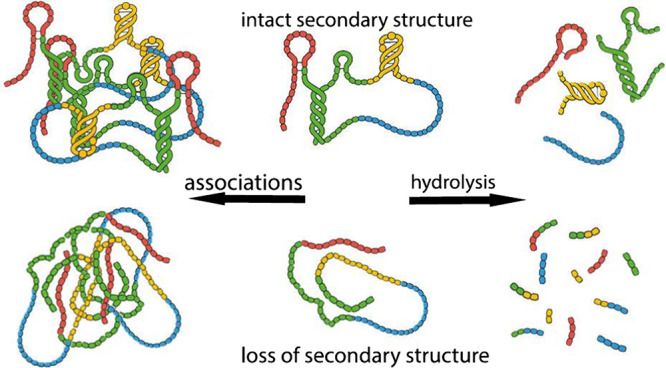

## Introduction

The emergence of RNA in medicine has generated a new focus on relating its molecular properties to its biopharmaceutics.^1^ In particular, the stability of RNA is critical in biopharmaceutic products. Whereas recombinant pharmaceutical biotechnology benefited from preceding decades of applied protein pharmaceutical sciences paralleled with milestones in structural biology, historical research on RNA oriented almost exclusively around its role in gene expression and fundamental cellular processes.^2^ While the principles of drug development and pharmaceutical quality control apply equally to RNA-based medicines to ensure purity, potency, strength, and identity, the innate molecular architecture of RNA is obviously quite different from existing peptide and protein-based drug products. This creates some special considerations for stabilization, characterization, and pharmaceutical control. Proteins are weak polyampholytes with low charge density incorporating both positive and negatively charged groups, whereas RNA is a strong polyanion at physiological pH due to its negatively charged phosphate ester backbone. The nucleobases of RNA also interact via H-bond and π–π-stacking interactions (HP effects). This charged backbone and HP effects create a different folding problem and result in some major distinctions in hydrodynamic and structural properties compared to proteins.^3,4^ The surrounding ionic cloud and spatial distribution of counterions becomes especially pertinent to the conformational thermodynamics of RNA.^5,6^ Another key attribute of mRNA is size, which can range from hundreds to thousands of nucleotides. Many mRNA vaccines currently in development use mRNA sequences in the length region of 2000–4000 nucleotides (the RNA molecule encoding a SARS-CoV-2 spike glycoprotein has a length of ∼4000 nucleotides with a molecular weight of ∼1.2 MDa). The sheer size of these therapeutic mRNA molecules adds structural complexity.^4,7^

Computational methods have been leveraged extensively to predict RNA secondary and tertiary structure, but it is acknowledged that these methods do not necessarily reflect how RNA molecules fold and function within cells, and it is suggested, by extension, within lipid nanoparticles (LNPs).^8,9^ These limitations originate from assumptions on which the energy calculations are based and because it is difficult to account for salt effects, which are central to the behavior of a strong polyelectrolyte.^10,11^

It is fundamentally important to realize that RNA has a vastly more complex space of secondary and tertiary structures than DNA, the latter being locked into very simple, elegant double helices, primarily B-DNA, but also A-DNA, Z-DNA, and triple^12^ and quadruple^13^ helices. As carriers of information, the stability of DNA structure is essential. In contrast, single-stranded RNA has the ability to form a very large number of secondary and tertiary structures. This ability to take on many varied structures enables RNA to perform functions beyond protein synthesis such as catalysis and structural scaffolding. A review of the complexity of higher-order structuring in RNA led to the term “structurome”, the complete set of possible RNA structures, and is analogous in meaning to a “genome” or “proteome”.^14^ Another review states that transitions between secondary structures can occur on the picosecond to hundreds of seconds time scales, and that these transitions need to be understood to elucidate how RNA can modify its behavior according to its surrounding physiological conditions.^15^

Another important aspect is the predominant mechanism for loss of RNA biological potency, which arises from its extreme susceptibility to strand cleavage by autohydrolysis.^16,17^ It takes only a single reaction event for an RNA molecule to lose potency. This special significance of RNA size, cleavage, and fragmentation places emphasis on measuring apparent molecular weight under pharmaceutically relevant conditions of temperature, time, and solvent composition. The problem of mRNA hydrolysis in the pharmaceutical context has been treated and the strategy proposed to increase the double-stranded region to protect against it.^18^ Light scattering is a versatile technique for the pharmaceutical analysis of large molecules and nanoparticles.^19–21^

Published results on the aggregation behavior of pure RNA under varying solution conditions are sparse. Tangential, but possibly related to the aggregation found in this work are studies of the effects of chiefly divalent salts on highly organized inter-RNA structuring, but not massive aggregation.^22,23^ Here, “massive aggregation” refers to nonspecific aggregates containing a large number of mRNA molecules. Concerning denaturants, such as Guanidine-HCl (Gd), most reports find good solubility of RNA in these.^24^ None has mentioned the type of massive aggregation found in this work.

This work reports on both massive aggregation of mRNA, mRNA, in ordinary aqueous solutions of both NaCl and Gd and, separately, thermally induced autohydrolysis and dissociation of low-level associations such as dimers. These separate results link the aggregation and autohydrolysis phenomena via the secondary and possibly tertiary structure of mRNA. mRNA is currently used in vaccines delivered by lipid nanoparticles.

To help address these aspects of mRNA solution behavior, this work uses time-resolved intensity light scattering intensity measurements, often referred to as static light scattering (SLS), as well as dynamic light scattering (DLS), to monitor both hydrolysis and aggregation.

It is shown here that RNA autohydrolysis is linked to its aggregation behavior under certain conditions. To map out the aggregation behavior, a device was employed, which allows real-time spectroscopic monitoring of the RNA during dialysis.^25^ With this device, it is possible to continuously monitor the addition to a macromolecular or colloidal solution of an agent, such as an electrolyte or denaturant, and then to subsequently monitor the reverse process. When aggregation or association occurs during dialysis, it is illuminating to make complementary time-dependent measurements of the aggregation/association process under fixed solution conditions. Here, dialysis using a monovalent electrolyte (NaCl) and a chaotropic agent (Gd) allows distinguishing electrostatic effects from attractive effects, such as π–π stacking, H-bonds, and the classical hydrophobic effect.

Intrinsic to the notion that secondary, tertiary, and possibly even quaternary RNA structure strongly influences its propensity for autohydrolysis and for colloidal aggregation is, as mentioned above, the vast complexity of these structures. Self-assembly of RNA into many different structural motifs has been extensively studied, and the field is mature enough that it is even used to create specific, synthetic RNA entities. The field is often termed “RNA nanotechnology”, or “RNA architectonics”.^26–28^ There are a great number of structural motifs, including stacks, helices, hairpin loops, bulges, internal loops, multiloops, joints, stems, pseudoknots, zippers, and kissing hairpins^29–31^ as well as long-range tertiary structures.^32,33^

A further phenomenon is dimerization or other low-level associations (trimer, tetramer, etc.) of RNA strands, whose occurrence and role have been investigated *in vivo*.^34–36^ The mechanisms and structures involved in dimerization are largely the same motifs as those that contribute to RNA secondary, tertiary, and quaternary structure.^37,38^

Given the vastness of the field of RNA structures, the current work does not seek nor is it able to identify which specific structures confer the resistance to hydrolysis and aggregation. Rather, the intent is to show extensive data on thermal, ionic strength, and denaturant effects on mRNA autohydrolysis, massive aggregation, and lower-level associations, such as dimers. The developed heuristic energy model is meant as an interpretive guide to the data. Others, working in computational and theoretical areas, might develop more detailed, accurate, and fine-grained models.

As to the central notion of this work, that secondary and tertiary structure partially protects mRNA against both autohydrolysis and association/aggregation, previous work in the field has already amply demonstrated RNA structural motifs aid resistance to autohydrolysis, but demonstrations for association/aggregation protection could not be found by the authors, which is not surprising because even the experimental occurrence of aggregation under monovalent salt and chaotropic agents has not itself been well documented. One computational group treated propensity for dimerization theoretically via a statistical physics approach by constructing appropriate partition functions and determining phase transitions from random coils at high temperatures to a “molten state” where many intra- and intermolecular structures are possible.^39^ Highly structured “superfolder” mRNA, with high levels of secondary structure, has been demonstrated to stabilize against hydrolysis.^40^ Double-strand RNA was found to be orders of magnitude more stable than single-strand RNA.^41^ A recent review focuses on enhancing secondary structure for decreasing hydrolysis,^42^ while another work delves into the mechanism of how secondary structure helps protect against hydrolysis and proposes strategies for reducing the frequency of unpaired bases.^18^

## Materials and Methods

### mRNA

Small quantities of mRNA were provided by Moderna, and the number of bases and molar masses for the 12 types is shown in Table 1. No further information, such as composition or sequences, was provided. The tailless mRNA did not have the usual poly­(adenine) tail and was only available in even smaller amounts. The poly­(adenine) tail has been implicated in many mRNA characteristics, including stability and enhanced translation and involves considerable complexity, with over 20 different protein subunits participating in cleavage and polyadenylation.^43,44^ The mRNA concentration was 5 × 10^–5^ g/cm^3^ in all experiments, unless otherwise indicated.

**Table 1:** mRNA Used

sample ID	#1	#2	#3	#4	#5	#6	tailless #7	tailless #8	tailless #9	#10	#11	#12
length	694	2854	2257	952	4101	1680	704	2020	1837	1680	1980	1980
mol. wt.	225,713	921,729	731,590	309,181	1,329,683	5.45 × 10^6^				537,600	633,600	633,600
% dimerization recovery @100 mM NaCl^a^	58	88	80	100		60				95	100	100

a Percent of initial *M*_w_ after heating and cooling ramp. See Figure 10a–d.

NaCl (Sigma-Aldrich, USP grade) and guanidine-HCl (Gd) (Sigma-Aldrich, ≥98%) were used as the electrolyte and chaotropic agent, respectively. Since NaCl and Gd both increase the conductivity of aqueous solutions, their concentration in fluid 1 (containing the mRNA) could be computed by continuously measuring the conductivity of fluid 2 (containing the circulating dialysate). Fluid 1 held 2.5 mL of mRNA solution and the volume of fluid 2 was 100 mL, meaning [Gd] = 150 mM in fluid 1 at the end of a complete dialysis against 6 M Gd and [NaCl] = 125 mM after dialysis against 5 M NaCl. Fluid 2 had 500 mL for reverse dialysis against buffer/water in fluid 2.

The buffer was 32.5 mM sodium acetate at pH 5.3 and was provided by Moderna. The pH of Gd-HCl in pure water ranges from 6.3 to 5.5 as the concentration is increased from 0.1 to 6M. Thus, a small increase in pH would be expected as the buffering capacity is depleted with the pH decreasing toward the starting pH of 5.3 as the Gd-HCl concentration increases in fluid 1.

The dialysis membrane was Sigma-Aldrich cellulose D9277 with ∼10,000 g/mol nominal molar mass cutoff.

The dialysis results are sometimes represented versus time and, in other instances, versus the concentration of electrolyte, either [NaCl] or [Gd]. It is sometimes more appealing to see the course of the dialysis in time, while at other times, representing the data versus [NaCl] and [Gd] can be more useful for analysis of the macromolecular changes occurring during dialysis and concentration regimes over which they occur.

### Static and Dynamic Light Scattering

A Brookhaven Instruments NanoBrook Omni instrument (Holtsville, New York) was used for dynamic light scattering (DLS). λ_0_ = 640 nm was the vacuum wavelength of the vertically polarized incident light, and light scattering detection was at θ = 90°, which gives the magnitude of the scattering vector:

$$q=\frac{4\pi n_{s}}{\lambda_{0}}\;\sin(\theta /2)=184,560\;{\mathrm{cm}}^{-1}$$ 1

where *n*_s_ = 1.333 is the index of refraction of water. The *z*-average self-diffusion coefficient, ⟨*D*_0_⟩_*z*_, and polydispersity index, *Q*, were obtained by standard second-order cumulant analysis. A second DLS instrument, a Brookhaven 90 Plus with λ_0_ = 640 nm, was also used.

⟨*D*_0_⟩_*z*_ (found at low concentration or by extrapolation to zero concentration) from DLS is used to compute the apparent *z*-average hydrodynamic diameter *D*_H,ap_, starting with the Stokes–Einstein equation for monodisperse spheres of hydrodynamic diameter *D*_H_ and self-diffusion coefficient *D*_0_:

$$D_{H}=\frac{k_{B}T}{3\pi \eta D_{0}}$$ 2a

where *k*_B_ is Boltzmann’s constant and η the solution viscosity. The dependence of η on *T* is corrected by using readily available physical data from NIST or CRC sources on aqueous NaCl and Gd solutions.

⟨*D*_0_⟩_*z*_ and *D*_H,ap_ are reciprocally related. Hence, *D*_H,ap_ is actually the reciprocal of the *z*-average inverse *D*_H_, which can differ considerably from the true *z*-average hydrodynamic diameter ⟨*D*_H_⟩_*z*_:

$$D_{H,\mathrm{ap}}=1/{\left\langle 1/D_{H}\right\rangle }_{z}$$ 2b

*Q* from the DLS autocorrelation function is the ratio of the second moment to the first moment squared of the polynomial expansion of the expansion of the logarithm of the electric field autocorrelation function:

$$Q=\frac{{\left\langle {D_{0}}^{2}\right\rangle }_{z}-{{\left\langle D_{0}\right\rangle }_{z}}^{2}}{2{{\left\langle D_{0}\right\rangle }_{z}}^{2}}$$ 2c

*Q* < 0.10 is generally considered low polydispersity, 0.10 < *Q* ≤ 0.25 medium polydispersity, and *Q* > 0.25 high polydispersity. An improved method for polydispersity determination has been proposed.^45^

Static light scattering (SLS) measurements were made on a Fluence Analytics (now Yokogawa Fluence Analytics, Houston, Texas) Argen device, equipped with 16 independent sample cells, each with its own adjustable temperature and stirring, incident laser source at λ_0_ = 660 nm, and detection at 90°. The Zimm approximation for polymers is

$$\frac{Kc}{I\left(q,c\right)}=\frac{1}{M_{w}}\left(1+\frac{q^{2}{\left\langle S^{2}\right\rangle }_{z}}{3}\right)+2A_{2}c$$ 3

where *c* is the polymer concentration (g/cm^3^), *I*(*q*, *c*) the absolute Rayleigh ratio (1/cm), *M*_w_ the weight-average molar mass, *A*_2_ is the second virial coefficient, ⟨*S* ^2^⟩_*z*_ is the *z*-average square radius of gyration, and *K* is an optical constant, which, for vertically polarized incident light, is

$$K=\frac{4\pi^{2}{n_{0}}^{2}{\left(\partial n/\partial c\right)}^{2}}{{\lambda_{0}}^{4}N_{A}}$$ 4

where $\frac{\partial n}{\partial c}=0.185\;{\mathrm{cm}}^{3}/g$ was used for the differential refractive index of mRNA in a low ionic strength aqueous solution. The values of *n*_0_ in aqueous solution as a function of [Gd] and [NaCl] are readily available from NIST and CRC sources, allowing the decreasing values of $\frac{\partial n}{\partial c}$ at higher [Gd] and [NaCl] to be computed.

For 90° detection, the term $\frac{q^{2}{\left\langle S^{2}\right\rangle }_{z}}{3}$ in eq 3 can be ignored if it is much less than 1. *D*_H,ap_ ∼ 26 nm was found for the second-largest mRNA, #2. ⟨*S* ^2^⟩^1/2^ is approximately related to *R*_H_ for a monodisperse ideal random coil by

$${{\left\langle S^{2}\right\rangle }_{0}}^{1/2}∼0.67D_{H}$$ 5a

With this approximation, for mRNA #2, $\frac{q^{2}{\left\langle S^{2}\right\rangle }_{z}}{3}=0.034$ , so that using 90° detection introduces only a 3.4% underestimate of *M*_w_, with the error less than this for the 10 smaller mRNAs in Table 1.

Separate determinations of *Kc*/*I*(*c*) versus *c* over a range of mRNA concentrations from 10^–6^ to 1.2 × 10^–4^ g/cm^3^ gave no measurable *A*_2_ so that the 2*A*_2_ *c* term in eq 3 is negligible over this range, including at 5 × 10^–5^ g/cm^3^, where all the dialysis and thermal ramps were carried out, as well as the measurements at fixed [NaCl], [Gd], and *T*.

Using the wormlike chain expression for the unperturbed ⟨*S* ^2^⟩_0_ in the coil limit

$${\left\langle S^{2}\right\rangle }_{0}=\frac{LL_{p}}{3}$$ 5b

yields an apparent persistence length *L*_p_^′^ = 23 nm (i.e., *L*_p_^′^ includes long-range excluded volume effects, whereas *L*_p_ does not), using a 0.6 nm contour length per nucleotide and the (nonideal) value of ⟨*S* ^2^⟩ = (17.3 nm)^2^ for mRNA #2. This apparent persistence length is of the order found in the literature, 20–30 nm, for mRNA with secondary structure.

### Real-Time Dialysis Monitoring

Real-time monitoring of SLS and DLS was achieved with a device, comprising a cap structure insertable into any 1 cm cuvette, which allows monitoring of dialysis and other membrane-mediated processes in any optical instrument, which accepts this type of standard cuvette, including UV/visible absorption, fluorimeter, and circular dichroism. The dialysis cuvettes were used directly with Brookhaven DLS and Fluence Argen. The device has been previously described.^25^

### Monitoring at Fixed [NaCl], [Gd], and *T* for Time-Dependent Processes

All methods involving the ramp of some physical parameter (e.g., temperature, pressure, and concentration of a molecule) can convolve the time scale of the ramp with the time scale of a sample’s physical change, if the two-time scales are on the same order of magnitude. For the changes in the physical characteristics to be meaningful as a function of the ramp parameter, the sample’s time-dependent processes must be much faster than the ramp rate, so that the system is instantaneously in equilibrium at each point during the temporal ramp. Accordingly, when there is evidence of time-dependent effects on the time scale of the dialysis, complementary time-dependent, nondialysis measurements were made at fixed [NaCl], [Gd], and *T*.

### On Terminology: Association, Colloidal Aggregation, and Reversibility/Semireversibility/Irreversibility

“Low-level associations” of mRNA, such as dimers and trimers, are linked to stable association states of mRNA and are sequence and structure dependent. Associations are generally reversible. “Aggregation” that involves many mRNA molecules in an aggregate and which can potentially grow further is termed “colloidal aggregation”, which leads to nonspecific clusters that lack the intermolecular “recognition” and specificity of dimers, trimers, etc., which are self-limiting in size and stable. “Aggregates” here can be fully reversible, semireversible, or irreversible. Data on these conditions are discovered using reverse dialysis. In the semireversible case, the aggregates partially dissociate but do not recover their original *M*_w_, perhaps being kinetically trapped. In the irreversible case, either the aggregates reach a large limiting case or, as happens more frequently, they continue to aggregate toward thermodynamically irreversible precipitation.

## Results

One caveat concerning the results is that the small, one-time amount of mRNA sample available made it impossible to perform all tests on all the mRNA samples. Hence, the results in the figures will show different mRNA types, depending on the availability of data for each phenomenon presented.

Many of the following results are significantly affected by secondary and possibly also tertiary structures of mRNA. A central notion of this work is that mRNA secondary structure provides some protection against both aggregation and autohydrolysis and that loss of secondary structure, either by a denaturant or temperature, leads to a higher propensity for these effects. On the other hand, aggregation can be treated consistently with a phenomenological energy-based model, presented in Analysis and Discussion, which does not take secondary structure explicitly into account and considers only the repulsive intermolecular electrostatic and attractive potentials, the latter potentially due to H-bonds, π–π stacking, and the classical hydrophobic effect (the entropic gain from releasing water molecules from hydrophobic surfaces). These attractive potentials are termed “HP” interactions in this work. A more detailed model will include the effect of secondary structure on the magnitudes of the HP interactions and how these can change aggregation profiles during dialysis. The results, including the thermal data, together with the phenomenological model point to π–π stacking and, to a lesser extent, H-bonds as the origin of the attractive potentials, not the classical hydrophobic effect. In fact, with reference to base-pairing, it was found that G-C pairing contributes almost no stabilization.^46,47^

### mRNA Aggregation Behavior during Dialysis against NaCl and Guanidine-HCl (Gd)

Figure 1a shows the *M*_w_/*M*_0_ for mRNA #1, initially in buffer, dialyzed against 6 M Gd, and then reverse-dialyzed against buffer. *M*_0_ is the native mass of the unassociated mRNA and *M*_w_ is the time-dependent weight-average value of all forms of mRNA in the solution at a given time, including monomers, dimers, trimers, and up to massive colloidal aggregates. *M*_w_/*M*_0_ = 1 is the initial state of the solution.

**Figure 1:**
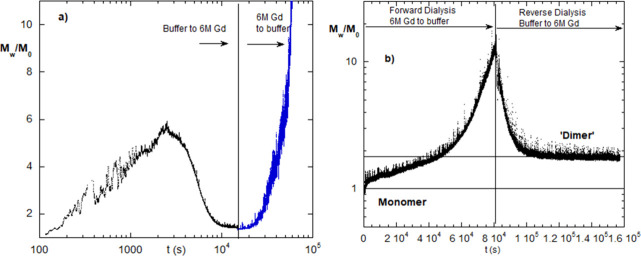
(a) Raw data vs time for mRNA #1 dialyzed against 6 M Gd, and reverse. The aggregation window is seen on forward dialysis, where aggregation sets in early, reaches a maximum, and then decreases. *T* = 25 °C. (b) Starting in 6 M Gd, mRNA #1 aggregation when dialyzing against water, then back to Gd. *T* = 25 °C.

A log-scale is used in Figure 1a for time to clearly see the “aggregation window” experienced by this mRNA early in the forward cycle as [Gd] increases. Over this window, the mRNA begins to associate after a few hundred seconds, reaches a maximum around 2000 s, and then decreases to nearly the starting value by 10,000 s, and stays flat for another 85,000 s (not shown). Then, upon reverse dialysis (the blue right-hand data in Figure 1a), after about 5000 s, there is an onset of massive colloidal aggregation. The Gd at first provides intermolecular electrostatic shielding, allowing the mRNA to aggregate via association of the nucleotides, but then these aggregates are destroyed at higher [Gd] and the mRNA reverts to a mass close to its original. However, the Gd has also caused loss of intramolecular secondary, and possibly tertiary, structure, so that, with all of these “exposed nucleotides”, the mRNA can undergo massive colloidal aggregation as the Gd is dialyzed away. “Exposed nucleotides” here refers to those not participating in secondary structure.

Figure 1b switches the order of the cycle of Figure 1a. Namely, mRNA #1 starts in a 6 M Gd solution and is dialyzed against buffer, which was truncated before reaching very low [Gd], which is why the aggregation window is not seen, and then reverse-dialyzed back to 6 M Gd. Following the above logic, mRNA is minimally self-associated in 6 M Gd and has also lost significant secondary structure. Hence, as Gd dialyzes away, HP interactions resume, along with aggregation. When dialyzed back to 6 M Gd, the HP interactions are again destroyed, along with the aggregation. The final portion of the reverse phase is labeled “dimer”, but has the quotation marks to indicate that it may not be a literal dimer just because *M*_w_/*M*_0_ = 2. A fortuitous value of the weight-average over monomers and low levels of dimers, trimers, tetramers, etc. could give the same experimental result, *M*_w_/*M*_0_ = 2. SLS does not furnish molar mass distributions. Whatever its origin, a final value of *M*_w_/*M*_0_ = 2 indicates that the aggregations are largely but not completely reversible.

Figure 2a contrasts the behavior of mRNA #4 when dialyzed against both NaCl and Gd. In the case of Gd, there is again an aggregation window, as was seen in Figure 1a for mRNA #1, but of much greater *M*_w_/*M*_0_ magnitude. For NaCl, there is simply a monotonic increase of aggregation and no aggregation window. This is because while NaCl shields intermolecular electrostatic repulsion, just as Gd did, it does not affect HP interactions, so that the aggregates do not fall apart at high [NaCl].

**Figure 2:**
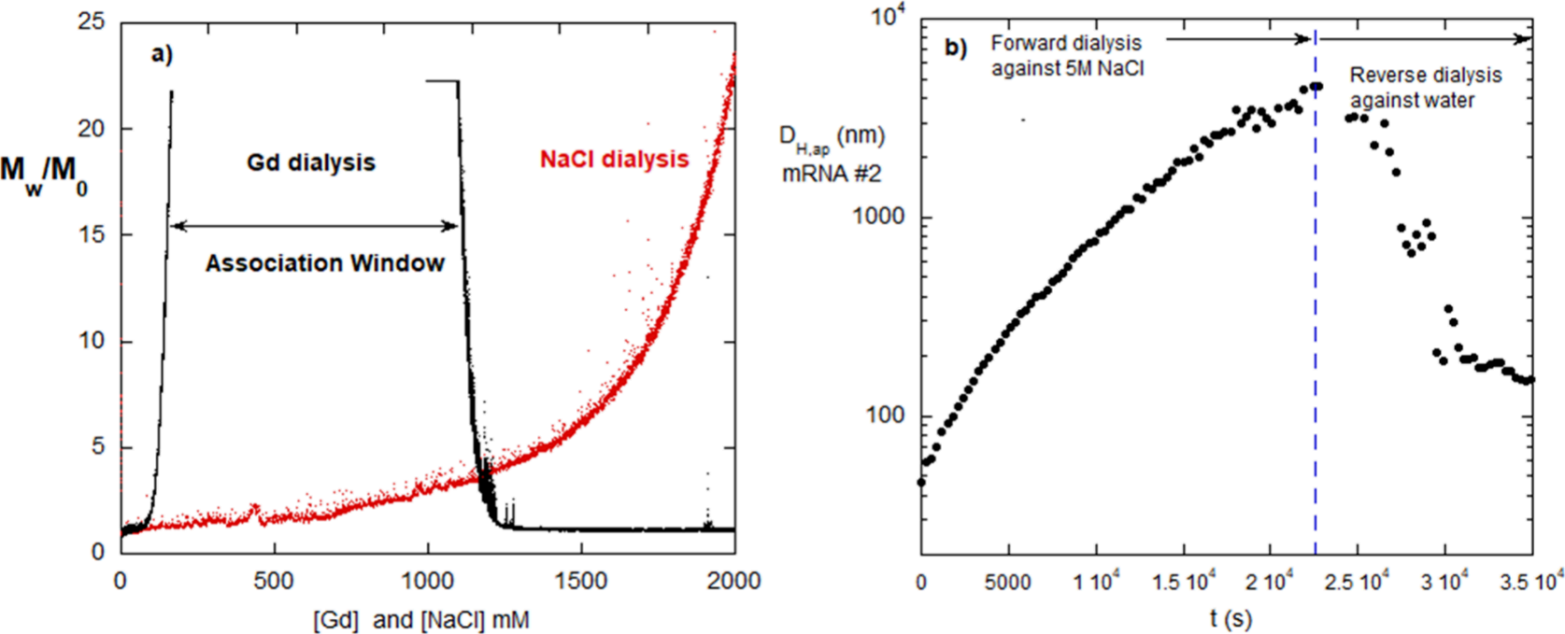
(a) Monotonic, semireversible behavior of mRNA #4 dialyzed against NaCl (red). Reversible aggregates formed over the aggregation window of mRNA #4 dialyzed against Gd (black). *T* = 25 °C. (b) Forward and reverse dialysis of mRNA #2 from buffer against 5 M NaCl and back to buffer. This shows semireversible behavior. *T* = 25 °C.

Figure 2b gives *D*_H,ap_ vs *t* during dialysis of mRNA #2 against 5 M NaCl. Similar to mRNA #4’s behavior in Figure 1a, it shows that aggregation occurs due to the electrostatic shielding and that these aggregates are only partly reversible upon reverse dialysis. According to the notion of protection against aggregation due to the secondary structure, the fact that there is high aggregation in NaCl suggests that this is due to nucleotides not involved in secondary structure, even in the mRNA native form. The fact that there is a significant residual population of irreversible aggregates when dialyzing back to buffer suggests that the HP interactions formed at high shielding are too strong to be undone by thermal energy once the NaCl is dialyzed away. This is consistent with the notion of π–π stacking as the main origin of the attractive potential.

### Fast Phase of mRNA Dissociation and Subsequent Slow Autohydrolysis

Figure 3 shows *M*_w_ of mRNA #6 vs time when held at *T* = 60 °C. There is a rapid and subsequent slow phase of diminishing *M*_w_. The initial fast dissociation is complete after 45 min and corresponds to dissociation of low-level associations of mRNA, such as dimers and trimers. At the end of this, fast-phase *M*_w_ is close to its native value in Table 1. After the fast dissociation, there is slow autohydrolysis of the mRNA over the next 7 h.

**Figure 3:**
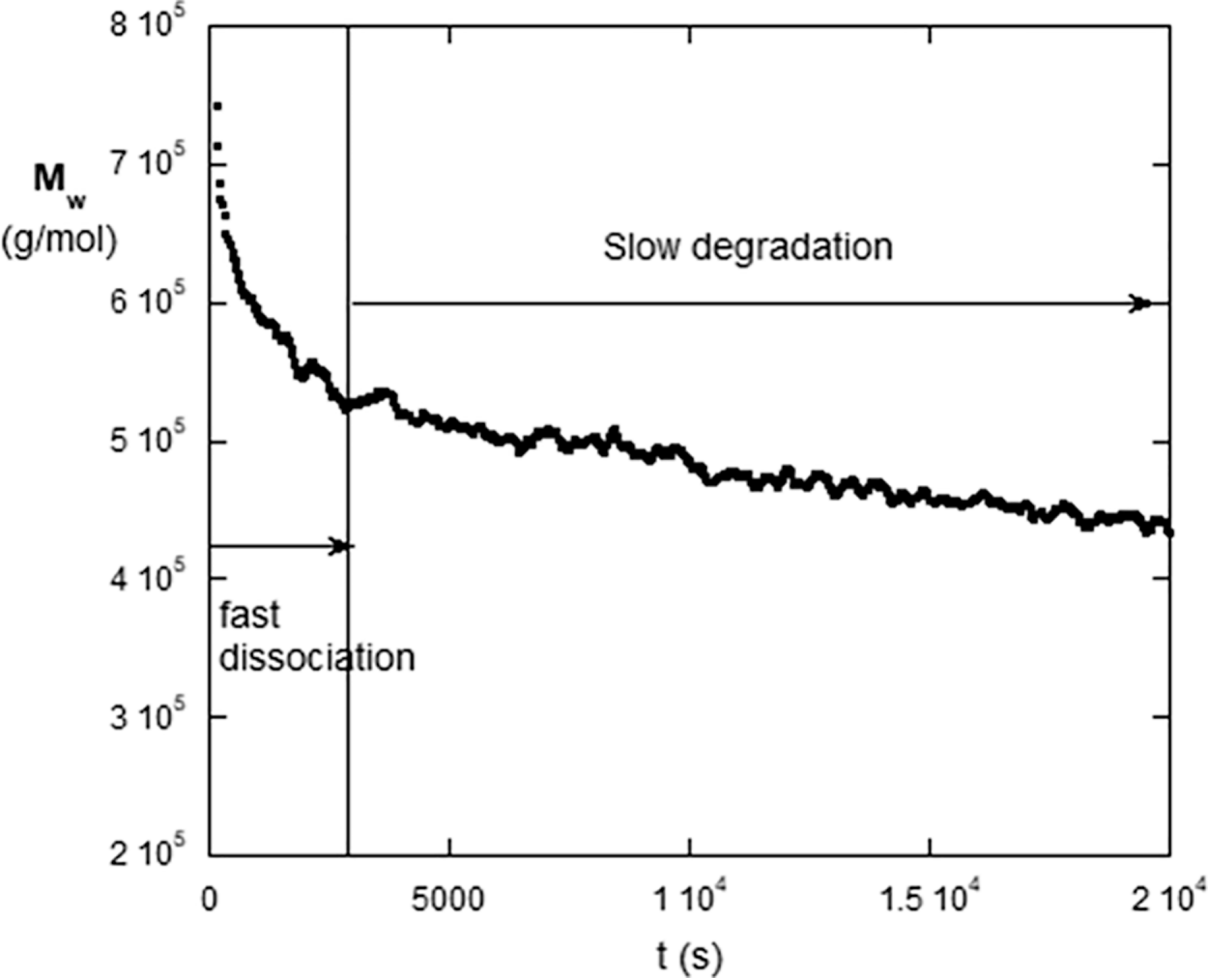
Initial fast phase of interchain dissociation of mRNA #6 at 60 °C, followed by a much slower autohydrolysis phase.

### Temperature-Dependent Hydrolysis Plateaus for *M*_w,final_/*M*_0_

Because autohydrolysis was still continuing after 7 h, autohydrolysis was subsequently carried out for 5 days for mRNA #12 at a series of temperatures: *T* = 40, 44, 48, and 55 °C, seen in the *M*_w_/*M*_0_ data in Figure 4a. Remarkably, at each temperature, a plateau in *M*_w_/*M*_0_ is reached, which decreases as *T* increases. Plateau data versus temperature are shown for other mRNA in Supporting Information.

**Figure 4:**
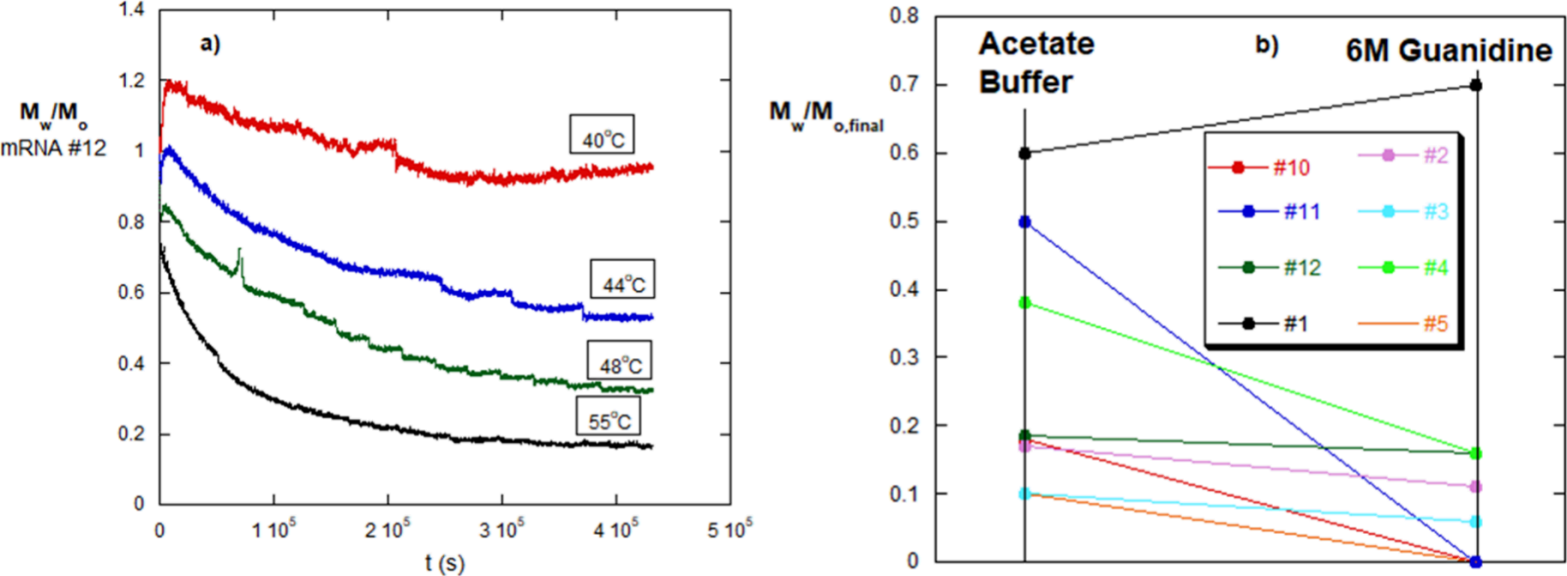
(a) Final *T*-dependent hydrolysis plateaus after 6 days of monitoring for mRNA #12. (b) Final *M*_w_ plateaus for various mRNAs in both the acetate buffer and 6 M Gd, at *T* = 55 °C; #1, #2, #3, #4, #5, #10, #11, #12.

The hypothesis on the dominant effect of secondary/tertiary structure posits that there are different strengths of the many types of secondary structure and that at each temperature monitored, a certain number of these secondary structures are undone, leaving more nucleotides exposed to autohydrolysis.

Following this notion, the use of denaturing Gd should undo more of the secondary structure, leading to lower plateaus. Figure 4b contrasts the final plateau values *M*_w,final_/*M*_0_ in the acetate buffer at *T* = 55 °C and in 6 M Gd, for eight mRNA types from Table 1.

In three cases, for #5, #10, and #11, virtually all secondary structure was destroyed, and *M*_w,final_/*M*_0_ is in the solvent scattering baseline, indicating that the mRNA was hydrolyzed down to very small fragments with no detectable residual scattering. At the very low mRNA concentration, 5 × 10^–5^ g/cm^3^, small fragments were not detectable by light scattering.

For mRNA #4, there was a large but incomplete drop in 6 M Gd, indicating that the Gd did destroy some secondary structure, exposing a larger number of nucleotides to autohydrolysis. For mRNA #2 and #3, there is a slight decrease in *M*_w,final_/*M*_0_, suggesting that only a small number of additional secondary structure was destroyed by the 6 M Gd. For mRNA #12, there is no meaningful drop in *M*_w,final_/*M*_0_, and for mRNA #1, there might have been a slight increase in the plateau.

The conclusion from Figure 4a,b is that the secondary structure protects against hydrolysis and that there is a wide variation in robustness against destruction of the secondary structure by Gd. In some mRNA, the secondary structure is destroyed enough that autohydrolysis leads to small, undetectable fragments, maybe including monomeric nucleotides. In other mRNAs, a portion of the secondary structure is destroyed, but not enough to bring autohydrolysis down to the small fragment or monomeric level. Finally, in two cases, the Gd did not enhance the autohydrolysis at all. It is further concluded that some secondary structures are so strong that they resist a strong denaturant, such as 6 M Gd; this can be termed “persistent secondary structure”.

### Thermal Phase Transition within the Gd Aggregation Window

Remarkably, aggregation within the aggregation window virtually ceases at and above a given critical temperature *T*_c_. Figure 5 shows the aggregation rates dropping by 3 orders of magnitude at *T*_c_ for mRNA #5 and by over an order of magnitude for #1, where *T*_c_ ∼ 40 °C for #5 and *T*_c_ ∼ 43 °C for #1. It is postulated that the HP interactions weaken with temperature and that the net energy will go positive at *T*_c_. The inset of Figure 5 shows the model-based net intermolecular energy as a function of temperature and the crossover from negative to positive. This is explained in Analysis and Discussion.

**Figure 5:**
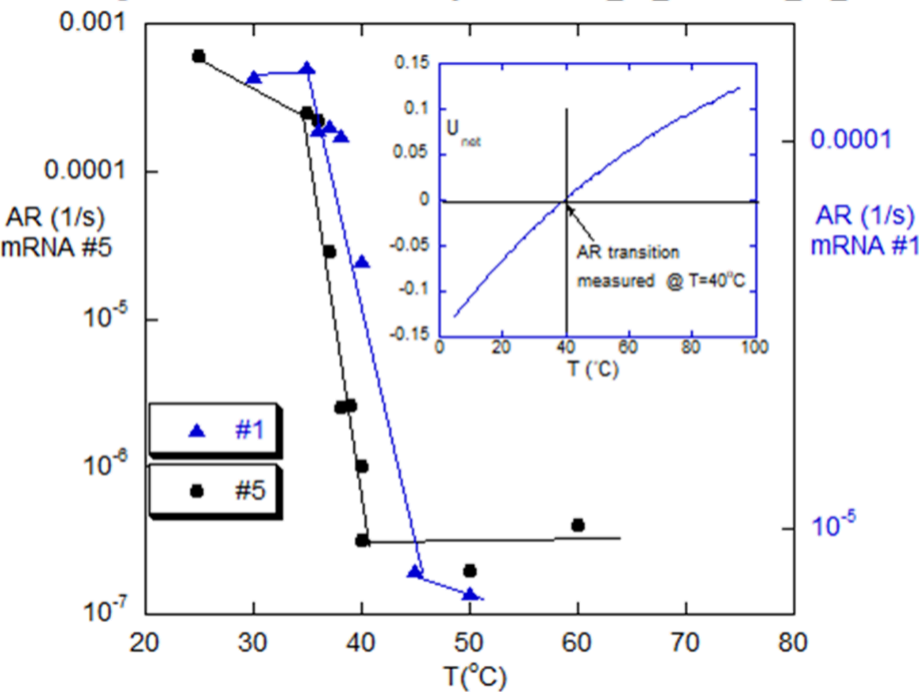
Abrupt drop in AR around 40 °C for mRNA #5 and around 43 °C for mRNA #1, in 2 M Gd, i.e., within the aggregation window. Note the two different y-scales. The inset shows computation of ⟨*U*_net_⟩ versus *T* and determination of *T*_c_ when ⟨*U*_net_⟩ = 0, for mRNA #5, using eq 22.

The aggregation rate (AR, in s^–1^) shown in Figure 5 is computed from the initial linear regime of *M*_w_(*t*)/*M*_0_ versus time:

$$\mathrm{AR}=\frac{d\left[\left(M_{w}\left(t\right)/M_{0}\right)\right]}{dt}$$ 6

### Hysteresis in Complete Temperature Ramp Cycles

Figure 6a shows *M*_w_/*M*_0_ for mRNA #2 during a temperature ramp, from 25 to 70 °C, in 100 mM NaCl. The up and down *T*-ramp each lasted 10^4^ s (see Supporting Information for both more hydrolysis/dedimerization data, such as Figure 4a, and *T*-ramp data, such as Figure 6a–c). The abrupt drop in LS at 58 °C for 100 mM NaCl is seen in all mRNAs with tails (the tailless mRNA was not tested, because of lack of material). This suggests this may be a general property of the mRNA samples, in that all mRNA samples tested—#1–#6 and #10–#12—yield the same transition temperature, *T*_t_, at a given [NaCl]. Also, all of the mRNA tested showed the “hump” starting around 50 °C and ending in the abrupt drop at *T* = 58 °C. The hump may be a reassociation before the final rapid dissociation and was found for all mRNA tested. The same behavior as *M*_w_/*M*_0_, including the hump and abrupt drop, was found for *D*_H,ap_ using DLS, which is included in Figure 6a.

**Figure 6:**
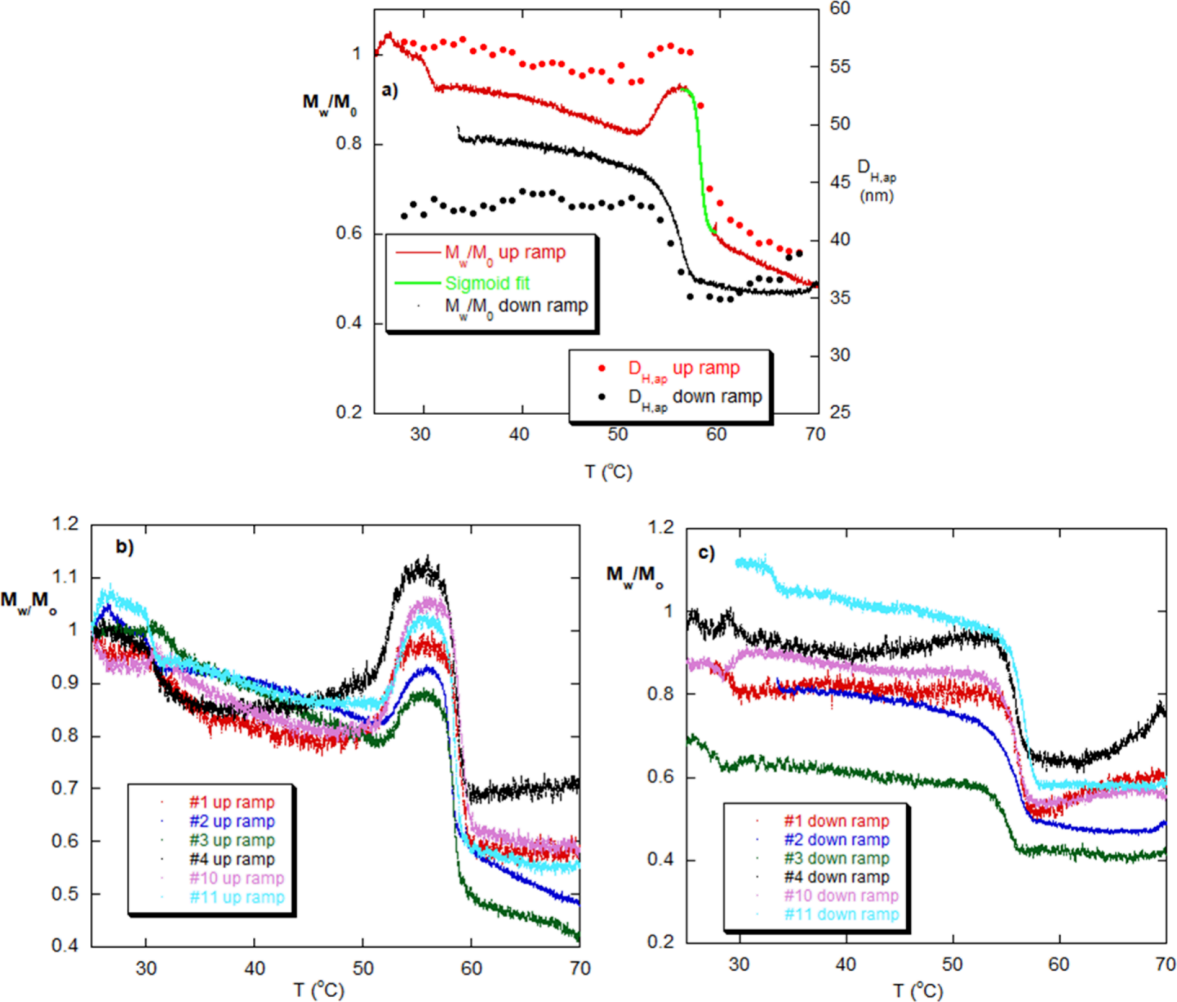
(a) Heating and cooling ramp for mRNA #2. The sigmoidal fit to eq 27 (green) is shown over the transition region. (b) Up ramps for mRNA #1–#4, #10–#11, showing the same transition phenomenon and the “hump” before it. There are qualitative differences between the trajectories leading to the hump. (c) Down-ramps for the data are shown in Figure 6b. Hysteresis is seen in several of these. Supporting Information gives more complete data on thermal hysteresis for several other mRNA types.

The 10^4^ s of the *T*-ramp is not enough time to cause significant hydrolysis over time, leading to the abrupt transition. Hydrolysis rates (HR, in s^–1^) are computed similarly to AR: HR = −d­(*M*_w_/*M*_0_)/d*t*. The negative sign ensures a positive value for HR. Typical hydrolysis rates are around 5 × 10^–6^s, e.g., from Figure 4a, normalized to *M*_w_/*M*_0_, so that over 10^4^ s, there should be less than 5% change in *M*_w_ due to autohydrolysis.

The final *M*_w_ values after the drop for all mRNA tested were roughly 1/2 the initial value. The abrupt decrease in scattering at *T* = 58 °C in Figure 6a, and for the other mRNA types, is identified with dissociation of low levels of association, resembling dimerization, because (i) the transition is very rapid and pronounced; (ii) the rapid drop occurs over about 300 s, while the hydrolysis time scales are a thousand times slower than this; (iii) the near 1/2 value in all mRNA types tested is consistent with dedimerization, or dissociation of a mix of low levels of associations; and (iv) the drop of *D*_H,ap_ from 57 to 39 nm in Figure 6a gives a ratio of 1.46. This suggests that both the dimer, and possibly other low-level associations, and the monomer resemble stiffened random coils, for which, in the nonfree draining limit, and with excluded volume, *D*_H,ap_ ∝ *M* ^0.58^. When a dimer falls apart, 57 nm should fall by 1.49, which is close to 1.46.

Figure 6a also shows the cooling ramp, which demonstrates a more gradual, rather than abrupt rise. While all the mRNA types had the same transition feature, the pattern of the hysteresis on the cooling ramp varied significantly. Supporting Information contains a series of figures, similar to Figure 6a–c, for various types of mRNA in Table 1, and also under different [NaCl]. In fact, the hysteresis curves vary widely among mRNA types. Some fully recover their initial *M*_w_, and this is indicated in Table 1 as “% recovery of initial dimerization”. There is no correlation between % recovery and number of base pairs. The hump, ubiquitous on the up-ramp, disappears in all of the down-ramps.

The increase of *M*_w_ upon cooling is likely redimerization or formation of a variety of low-level associations. The fact that it does not fully recover the initial *M*_w_ may indicate that not all dimers are between the same set of bases on associating strands and that some are blocked from reforming. Below about 25 mM NaCl, the abrupt reduction no longer occurs at the temperatures measured.

Figure 6b shows there is a variety of trajectories of *M*_w_/*M*_0_ leading up to the transition, which, by this conjecture, is linked to the many types of dimers formed by each particular mRNA. In the one instance where mRNA #1 was reramped back up after the cooling ramp, the hump was lost, *T*_t_ was 1 °C lower than on the first up-ramp, and the trajectory up to the transition was flat (see Figure S7a). This suggests a sort of “annealing” of dimers in the first ramp cycle, leaving less of a variety of dimers in the “annealed” mRNA solution. In fact, even in the single-*T*-ramp cycles, the trajectory from 70 °C back to 25 °C is generally flatter than the up-ramp, and the hump has disappeared. This is possibly related to the annealing process.

Figure 6c shows the temperature down-ramp for the data in Figure 6b. There is a wide variety of return paths, leading to different levels of hysteresis for each type of mRNA. Table 1 gives the percentage recovery of the initial *M*_w_/*M*_0_ after the hysteresis cycle is complete.

Figure 7 shows that *T*_t_ decreases as the concentration of NaCl increases. This result is counterintuitive, because it seems the two negatively charged mRNA strands in the dimer should repel each other and destabilize the dimer, so that increasing IS should reduce the repulsion and stabilize the dimer. A conjecture to explain this experimental result is that the mRNA strands conform their secondary/tertiary structure to each other to lower the dimerization energy and stabilize the dimer, and that they are in conformations different secondary structures in monomers. When a certain transition temperature *T*_t_ = *T*_t_([IS]) is reached, there is a conformational change in the two mRNA molecules that causes them to separate over a narrow temperature window centered at *T*_t_. With increasing IS, the shielding between charges on opposite strands may make each strand more conformationally flexible, allowing each to drop to its own monomeric conformational energy, without electrostatic interference from the other strand. By the same token, as *T*_t_ is approached, the conformational flexibility resulting from the electrostatic shielding allows reassociation of mRNA monomers to form in combinations available at a given *T* and ionic strength. Soon after the hump in the temperature up-ramp, however, the conformational flexibility is great enough to allow the two mRNA strands to separate and fall into their minimum monomeric energy state.

**Figure 7:**
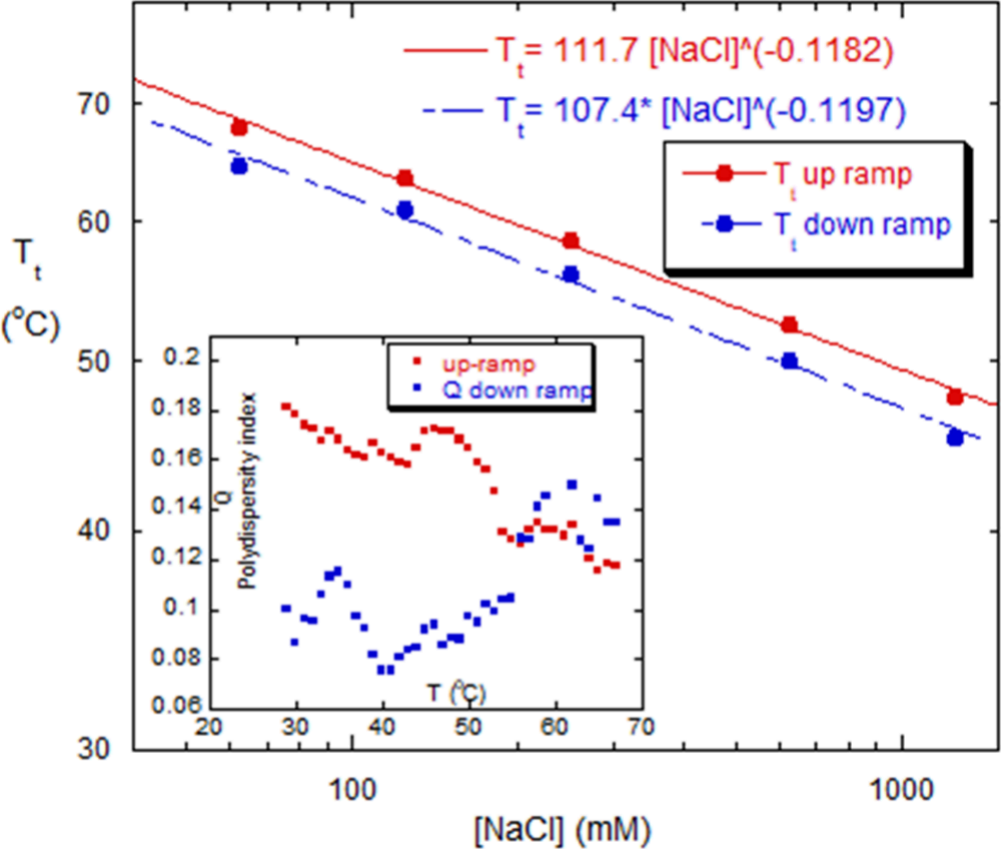
*T*_t_ vs [NaCl]. *T*_t_ for up- and down-ramps for mRNA #1. The inset shows the DLS polydispersity index *Q*, for the *T*-ramp data of Figure 6a, for mRNA #2.

The inset of Figure 7 is the DLS polydispersity index *Q* (eq 2c) for the *T*-ramp data of mRNA #2 in Figure 6a. It shows polydispersity decreasing during the up-ramp and includes the hump seen in both *M*_w_/*M*_0_ and *D*_H,ap_. This implies that the population becomes measurably less polydisperse as dimers and other low-level associations dissociate, and that polydispersity remains low as new dimers form on the down-ramp. This trend supports (i) the assertion that the decrease in both *M*_w_/*M*_0_ and *D*_H,ap_ in Figure 6a is due to dedimerization, or dissociation of low-level associations, rather than hydrolysis, as hydrolysis would lead to higher *Q* as the mRNA breaks into fragments, and (ii) the hump in the up-ramp might well be due to some fresh dimerization prior to *T*_t_ as the mRNA strands gain more conformational flexibility as *T* increases. The empirical power law exponent for *T*_t_ vs [NaCl] is around −0.12 for both the up and down-ramps.

### Results on Tailless mRNA Follow General Trends of Tailed mRNA, with Some Exceptions and Qualitative Differences

mRNA #7–#9 lacked the poly­(adenine) (poly­(A)) tail, which is related to several mRNA biological properties, including its stability, protection from certain enzymes, and interactions with certain poly­(A) binding proteins that make translation more efficient. So, it is of interest to see if the lack of the poly­(A) tail markedly affects the above phenomena concerning thermal, ionic, and chaotropic stability. Very little sample was available for study, but the trends found include the following: An aggregation window was found for dialysis against Gd, but it appears significantly broader than for the tailed mRNA, suggesting that susceptibility to massive colloidal aggregations is greater for tailless mRNA. The AR followed the window, similar to tailed mRNA. The sharp drop in AR at a critical temperature was found at around 40 °C. The aggregates were at least partially reversible under Gd and NaCl dialysis cycles. The tailless mRNA had discrete thermal degradation plateaus of *M*_w_/*M*_0_, like those in Figure 4a,b.

## Analysis and Discussion

### Phenomenological Interpretive Model for Intermolecular Polyelectrolyte and H-Bond/Hydrophobic/π–π Stacking Effects: the E/HP Model

The motivation for the following electrostatic/hydrophobic model (E/HP model) is to provide an energetic framework for consistently interpreting the various results. More complete, accurate, computationally based treatments are appropriate but beyond the scope of this work. The phenomenological model for interpreting the trends combines repulsive interactions between polyelectrolyte chains, (E), and associative interactions due to H-bond, hydrophobic, and π–π stacking potential energies (the latter are also referred to as “π–π interactions” and “aromatic interactions”); *“*HP” is used as a shorthand for H-bond, hydrophobic, and π–π stacking. These latter three effects include making and breaking of H-bonds, the classical hydrophobic effect due to gain in water entropy as it is released from unfavorable hydrophobic groups, and attractive interactions between the π-bonds, which are fundamentally quantum mechanical in nature. There are no attractive electrostatic polyampholyte interactions in this model, as there would be if polyampholytes (e.g., proteins) were involved,^25^ since RNA is a strong polyanion.

The model was first introduced in reference^25^ and is summarized here and expanded to include temperature effects on massive aggregation, and, separately, effects on dissociation of low-level associations, such as dimers and trimers. Similar potentials will be used but within an intramolecular context, treating the dimer as a single entity, divisible into two strands.

There are significant differences between denaturation of proteins and their subsequent aggregation and denaturation of RNA and its subsequent aggregation. In the former case, it is chiefly the classical hydrophobic effect—the increased entropy of water when released from hydrophobic contacts—that comes into play when proteins are denatured and their hydrophobic amino acids are exposed to the aqueous environment and hence aggregate to increase water entropy. Protein aggregates, once formed, are seldom reversible. While there is evidence of π–π stacking between aromatic amino acids, and their breaking by chaotropic agents, in the case of RNA, it is chiefly π–π stacking and H-bonds that allow nucleobases to associate. π–π stacking is often considered a form of hydrophobic interaction, because it also leads to exclusion of water and water’s entropy increase, although the mechanism is quite distinct and, as mentioned, quantum mechanical. π–π stacking generally involves interactions between aromatic rings via overlap of π orbitals. This distinction is important when temperature effects are considered, because the experimental evidence here is that the HP interactions weaken as temperature increases (Figures 5 and 6a–c). It is known that the hydrophobic interactions in proteins increase with temperature.^48–50^ In contrast, it is known that π–π stacking decreases with temperature.^51,52^ Furthermore, π–π stacking decreases more rapidly than H-bonds with increasing temperature,^53^ so it is surmised here that π–π stacking dominates in terms of the formation of associations, with a lesser role played by H-bonds. The E/HP model allows one to interpret abrupt changes in aggregation rates and dimerization at critical temperatures.

The E/HP model seeks plausible forms of net potential energies under different interplays of E, simple electrolytes (here, NaCl), and HP for chaotropic agents (here, Gd). When the net potential energy is positive, no association or aggregation is expected. Furthermore, if aggregates or associations are initially present, then the positive potential energy may lead to their dissociation. If the net potential energy is negative, reversible associations or irreversible or semireversible aggregates may form.

The use of NaCl and Gd in the dialysis helps to separate which effects, E or HP, are dominant over different ranges of concentration. The intent of the model is to guide plausible, consistent interpretations of the many very different data trends and not to provide a detailed theoretical model or simulation. Future efforts can be sought to refine the simplifications and approximations used here. Such efforts can also seek absolute values for potential energies, which are kept in arbitrary units here. As a reference for magnitudes of the energies, the electrostatic potential energy between two unscreened elementary charges separated by a distance of 1 nm in water (dielectric constant ∼ 80) at *T* = 300 K is approximately 2.9 × 10^–21^ *J* ≈ 0.018 eV = 0.7*k*_B_ *T*, where eV = electronvolt.

The model allows macromolecules to interact by both effects E and HP. The polyelectrolyte (E) term considers that the macromolecules each have a net charge and hence repulsive interactions, given by a positive screened electrostatic potential energy, *U*_E_. The model then posits a negative interchain potential energy due to HP effects, *U*_HP_.

For a nonchaotropic salt, such as NaCl, only *U*_E_ varies as ionic strength changes. *U*_HP_, which is negative, remains constant. For a chaotropic agent, e.g., Gd, *U*_HP_ decreases as [Gd] increases.

As a first approach, the mRNA molecules are considered as spheres of radius *R* and net charge *q*, uniformly distributed on the surface. The screened electrostatic potential of a charge ϕ_E_(κ, *r*) at distance *r* is given (in volts) by

$$\phi_{E}(\kappa ,r)=\frac{q}{4\pi \varepsilon (1+\kappa R)}\frac{e^{-\kappa (r-R)}}{r},,r\geq R$$ 7

where *r* is the distance from the center of the charge and κ is the Debye screening parameter, given by (in MKS units):

$$\kappa ={\left(\frac{2\rho_{0}ze^{2}}{\varepsilon k_{B}T}\right)}^{1/2}$$ 8

where ρ_0_ is the bulk charge density (C/m^3^) of added electrolyte, Gd, or NaCl in this work (ρ_0_ is the charge density of the positive charge, which of course is equal and opposite in sign to the negative charge density that reflects electroneutrality in the simple bulk electrolyte solution); *e* is the elementary charge; *z* is the valence for symmetric electrolytes (*z* = 1 for Gd and NaCl), ε = ε_0_D, where ε_0_ = 8.85 × 10^–12^ C^2^/N m^2^ is the permittivity of free space and *D* is the dielectric constant of the solution (∼80 for H_2_O at STP); *k*_B_ is Boltzmann’s constant (1.38 × 10^–23^ J/K); and *T* is the temperature in Kelvin. Note that κ is very weakly dependent on *T* near 25 °C; it decreases only 8% from 25 to 75 °C. ρ_0_ (C/m^3^) is proportional to the ionic strength [IS] (mol/L) of the electrolyte:

$$\rho_{0}=1000N_{A}ze[\mathrm{IS}]$$ 9

Since Gd and NaCl are monovalent, [Gd] and [NaCl]­are used for [IS] in their respective cases.

The expression for the screened electrostatic potential energy between two finite size spherical charges, *U*_E_ is much more complex^54,55^ than eq 7, but a useful approximation is to consider the mRNA molecules far enough apart that two molecules interact essentially as point charges (the solutions were at 5 × 10^–5^g/cm^3^, so for *M* ∼ 10^6^g/mol, the average spacing between mRNA molecules is about 300 nm, i.e., much greater than *R*_mRNA_ ∼ 30 nm). With this,

$$U_{E}(r)\approx \frac{q^{2}}{4\pi \varepsilon (1+\kappa R)}\frac{e^{-\kappa (r-R)}}{r},,r\geq R$$ 10a

This electrostatic potential energy is next Boltzmann-averaged over all of the space between charges. Because of the assumed spherical symmetry of the interaction, the solid angle over all space ∫dΩ = ∫sin^2^ θ dθ dϕ = 4π cancels in the numerator and dominator of the average, leaving just the integral over *r*, where 2*R* is taken as the distance of closest approach:

$$\langle U_{E}\rangle \approx \frac{\int_{2R}^{\infty }e^{-U_{E}(r)/k_{B}T}U_{E}(r)r^{2}\;\mathrm{dr}}{\int_{2R}^{\infty }e^{-U_{E}(r)/k_{B}T}r^{2}\;\mathrm{dr}}$$ 10b

This involves several integrals that do not have closed-form solutions and need to be solved numerically. Because distance has been averaged out in the potential energy and because the term e^–κ*r*^ in eq 10a will survive the integrations in eq 10b, the average electrostatic potential energy can be represented by

$$\left\langle U_{E}\right\rangle \left(\left[\mathrm{IS}\right]\right)={\left\langle U_{E}\right\rangle }_{0}e^{-\beta \sqrt{\left[\mathrm{IS}\right]}}$$ 10c

where ⟨*U*_E_⟩_0_ is the positive potential energy between two unscreened charges of the same sign (i.e., between two polyelectrolyte chains) averaged over space. β subsumes the factors connecting κ and [IS] in eqs 8 and 9, and the Boltzmann averaging over distance.

Now, ⟨*U*_HP_⟩([IS]) represents the HP associations, averaged over the interchain space. Because HP effects are usually cooperative in biomacromolecular systems, it is common to use sigmoid functions when modeling processes involving them,^56,57^ including the Hill equation,^58,59^ and the logistic function.^60^ Both functions should capture the HP effects, and a plausible sigmoidal ⟨*U*_HP_⟩([Gd]), the average HP potential based on the logistic function is

$$\langle U_{\mathrm{HP}}\rangle ([\mathrm{Gd}])=B-\langle U_{\mathrm{HP}}\rangle_{0}\frac{1}{1+e^{-\gamma ([\mathrm{Gd}]-[\mathrm{Gd}{]}_{1/2})}}$$ 11a

where ⟨*U*_HP_⟩_0_ is a negative potential energy, which is independent of [NaCl]. For NaCl, ⟨*U*_HP_⟩ is constant:

$$\langle U_{\mathrm{HP}}\rangle ([\mathrm{NaCl})]=\langle U_{\mathrm{HP}}\rangle_{0}<0$$ 11b

where the negative ⟨*U*_HP_⟩_0_ is the hydrophobic potential energy at [Gd] = 0, [Gd]_1/2_ is the concentration of Gd at which the sigmoidal energy is at its half-value, and γ controls the rate at which increasing [Gd] diminishes HP interactions. *T* = 25 °C was held constant during dialysis, so the *T*-dependence of *U*_HP_ is not explicitly shown here but is treated below. *B* is a constant:

$$B=\langle U_{\mathrm{HP}}\rangle_{0}\frac{2+e^{\gamma [\mathrm{Gd}{]}_{1/2}}}{1+e^{\gamma [\mathrm{Gd}{]}_{1/2}}}$$ 12

which ensures that

$$\langle U_{\mathrm{HP}}\rangle ([\mathrm{Gd}]=0)=\langle U_{\mathrm{HP}}\rangle_{0}$$ 13

The average net energy of the system, ⟨*U*_net_⟩([Gd]) and ⟨*U*_net_⟩([NaCl]), is just the sum of the electrostatic and hydrophobic potentials. The net potential energies are then

$$\langle U_{\mathrm{net}}\rangle ([\mathrm{IS}],[\mathrm{Gd}])=\langle U_{E}\rangle ([\mathrm{IS}])+\langle U_{\mathrm{HP}}\rangle ([\mathrm{Gd}]),\mathrm{for}\;\mathrm{Gd}$$ 14a

$$\langle U_{\mathrm{net}}\rangle ([\mathrm{IS}]])=\langle U_{E}\rangle ([\mathrm{IS}])+\langle U_{\mathrm{HP}}\rangle_{0},\mathrm{for}\;\mathrm{NaCl}$$ 14b

­[IS] is used for the ⟨*U*_E_⟩ term in eqs 14a and 14b because NaCl and Gd have similar electrostatic screening properties. Equation 14b differs from eq 14a because NaCl does not affect ⟨*U*_HP_⟩, and the HP effect remains as the constant ⟨*U*_HP_⟩_0_. The temperature dependence of ⟨*U*_HP_⟩ = ⟨*U*_HP_⟩([Gd], *T*) is treated below and explains the sharp transition temperature for aggregation rates seen in Figure 5.

The behavior of ⟨*U*_net_⟩ in eqs 14a and 14b determines whether interacting macromolecules dissociate or remain dissociated (⟨*U*_net_⟩ > 0), associate, or aggregate (⟨*U*_net_⟩ < 0). Hence, the condition that ⟨*U*_net_⟩ = 0 defines the existence of a bound (aggregate) state between mRNA molecules. In developing biologic drug formulations, the concentration regimes of electrolytes and other excipients can be determined so as to optimize stability of the formulations.

### Energetics of Aggregation in the Gd Window versus Monotonic Aggregation Increase in NaCl

Figures 1a and 2a show the aggregation window for mRNA nos. 1 and 4, respectively, when dialyzed against Gd. In order to more closely assess the mRNA behavior over the entire range of [Gd], complementary iso-ionic strength measurements were made. Figure 8a shows the behavior of mRNA #5 at selected Gd concentrations from 0 to 6 M Gd. At 0 and 0.1 M Gd, there is no intermolecular aggregation, but this sets in by 1 M and lasts until 3 M. By 4 M, aggregation occurs but very slowly. At 6 M Gd, there is no aggregation, showing the high [Gd] side of the window.

**Figure 8:**
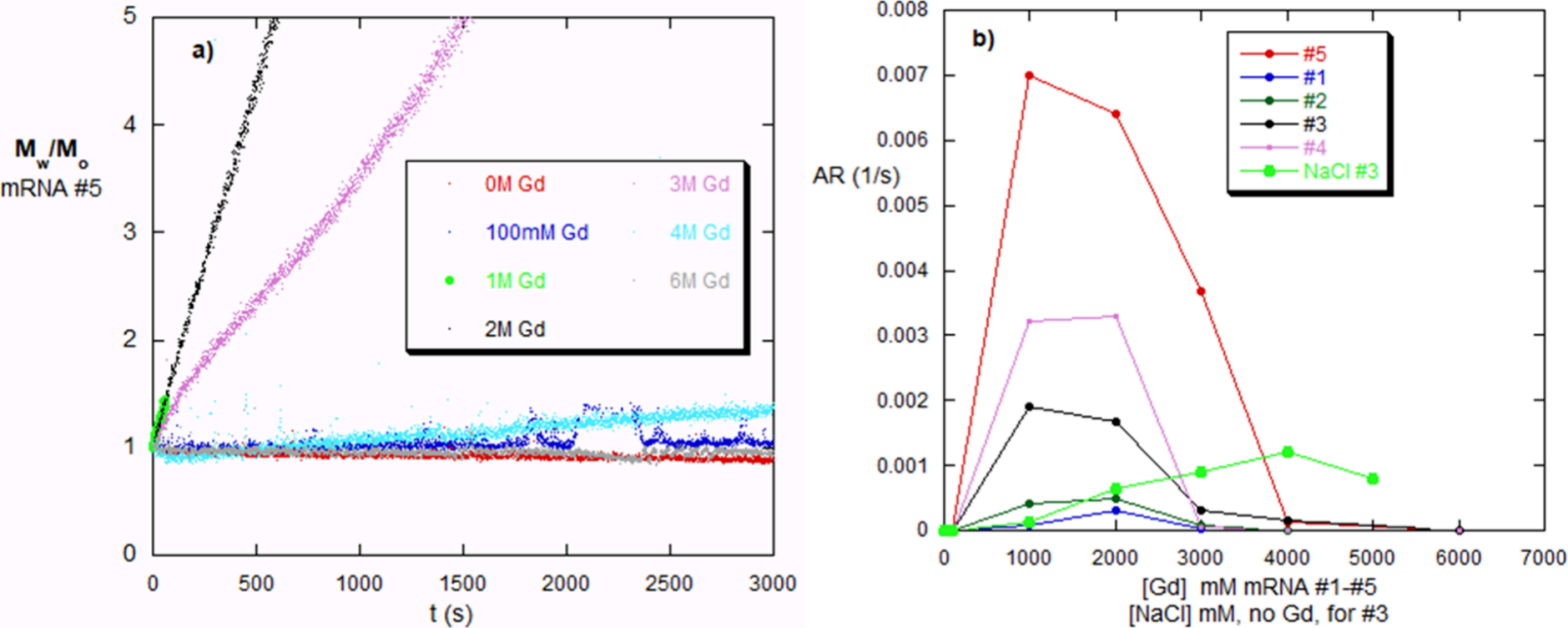
(a) *M*_w_(*t*)/*M*_0_ for mRNA #5 for discrete values of [Gd] from 0 to 6 M. (b) AR obtained according to eq 6 from the data such as in panel (a); RNA nos. 1–5 Also shown is AR versus [NaCl] for mRNA #3, for which no AW is found.

Figure 8b shows the aggregation rates, AR, using eq 6, for mRNA #1–#5. These map out the same type of window as those in Figures 1a and 2a, with the window occurring over approximately the same range of [Gd] for these mRNAs. The magnitudes of the AR, however, vary widely among the mRNA types, which can serve as another indicator of the robustness of its secondary/tertiary structure. Also shown in Figure 8b are the AR values for mRNA #3 for increasing values of [NaCl]. These ARs also correlate well with the monotonic NaCl aggregation results in Figure 2a,b.

The reason for the strong correlation between AR and the dialysis aggregation window is intuitively plausible; if a system self-associates, it does so at a certain rate. The higher the propensity for aggregation, the higher the AR. In the E/HP model, reversible association, or irreversible aggregation, can occur when the net energy in eq 14a or 14b is negative. If the barrier energy, which is different than ⟨*U*_net_⟩, varies in tandem with ⟨*U*_net_⟩, then AR will be correlated directly with ⟨*U*_net_⟩. The rate at which aggregation occurs, if Arrhenius-like, should then increase exponentially with the magnitude of the net-negative energy, if the barrier energy and ⟨*U*_net_⟩ are directly linked. This is found in Figures 8a,b, 9b, and 10b.

**Figure 9:**
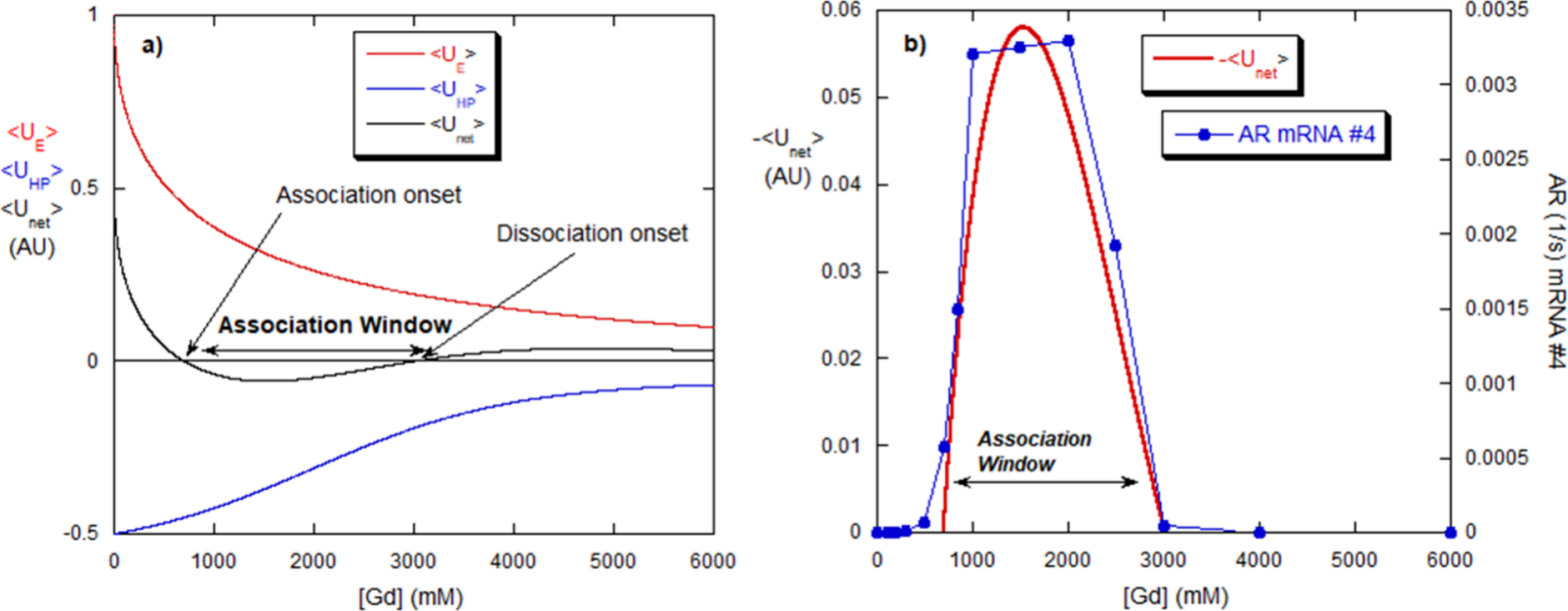
(a) Computed values for the net and component energies versus [Gd] are from the model. (b) Negative portion of net energy in Figure 4a, made positive, and superposed on the AR for mRNA #4, found at various [Gd]. The model computation captured the aggregation window.

**Figure 10:**
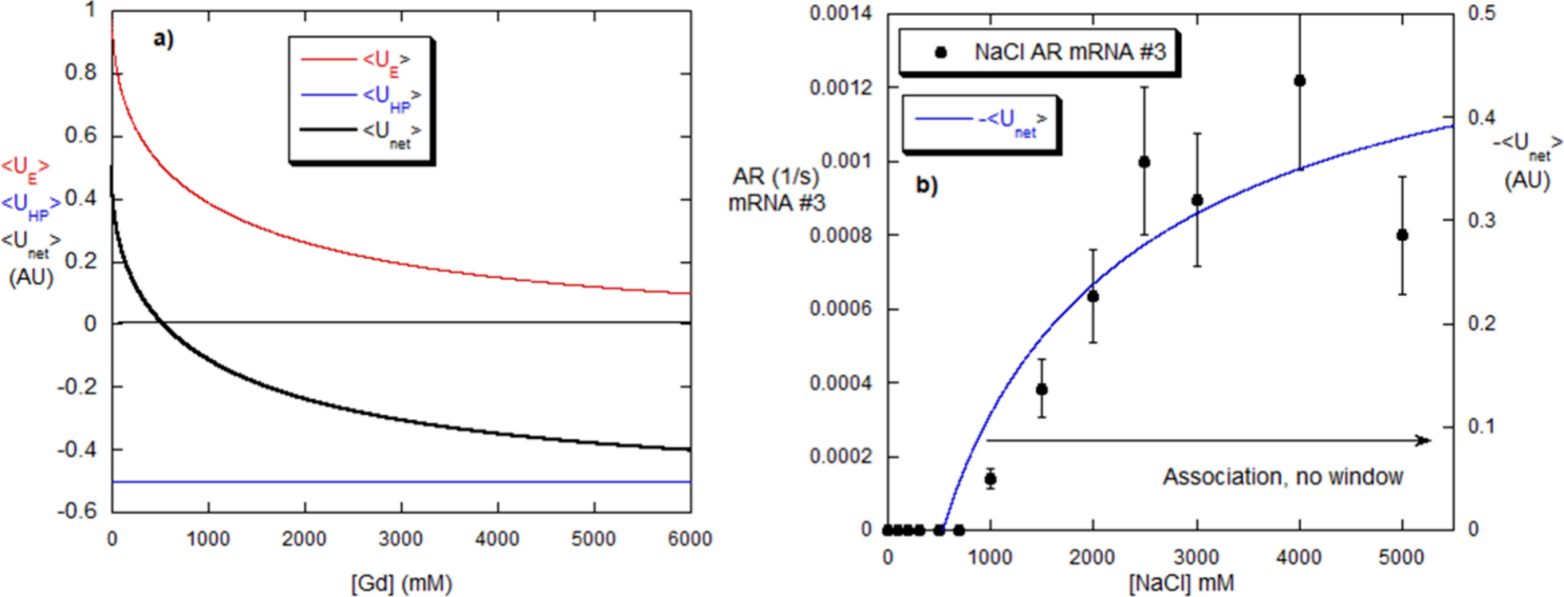
(a) Computed energies versus [NaCl] for mRNA. (b) The AR for mRNA #3, determined from separate measurements at constant [NaCl], follows the model computation, where the computed negative energy is made positive, similar to Figure 9b.

The Gd aggregation window results of Figures 1a and 2a and its correlation with the AR window seen in Figure 8b can now be analyzed with the E/HP model, as well as the monotonic NaCl results in Figures 1a and 2a and their correlation with the AR data in Figure 8b.

The model anticipates the existence of the aggregation window for Gd over a range of parameters. Figure 9a shows the ⟨*U*_*E*_⟩([Gd]), ⟨*U*_HP_⟩([Gd]), and ⟨*U*_net_⟩([Gd]) from these parameters: *U*_E,0_ = 1 (AU); *U*_HP,0_ = −0.5 (AU); [Gd]_1/2_ = 2000 mM; β = 0.03; γ = 0.001; *Β* = –0.05237. The net energy ⟨*U*_net_⟩([Gd]) goes negative over the range of [Gd] from 0.6 to 3 M [Gd]. In Figure 9b, the negative energy portion of Figure 9a has been excised and turned positive in order to compare it with AR over the aggregation window. Remarkably, the E/HP model contains the aggregation window found by dialysis and confirmed by the complementary iso-[Gd] measurements in Figures 8b and 9b. Again, the fact that the ARs, which are kinetic values, are strongly correlated with the negative potential energy suggests that AR and the potential energy shift in tandem as [Gd] changes.

The E/HP model’s eq 14b predicts that the aggregation of mRNA versus [NaCl] should increase monotonically. Figure 10a shows ⟨*U*_E_⟩, controlled by the ionic strength provided by NaCl, and ⟨*U*_HP_⟩_0_ = constant (negative), since NaCl does not affect HP interactions. ⟨*U*_net_⟩ becomes negative at intermediate [NaCl] and then decreases and becomes further negative as [NaCl] increases. This is a result of the constancy of ⟨*U*_HP_⟩ versus [NaCl]. The parameters are the same as those used in Figure 9a—*U*_E,0_ = 1 (AU); *U*_HP,0_= −0.5 (AU); β = 0.03, except that γ, *B*, and [Gd]_1/2_ do not appear in eq 11b for ⟨*U*_HP_⟩([NaCl]).

Figure 10b shows the AR determined by monitoring *M*_w_/*M*_0_ versus time at fixed values of [NaCl]. The monotonic increase of aggregation versus [NaCl] is contained in the E/HP model. The fact that AR follows the increase in net-negative energy shows that the more negative ⟨*U*_net_⟩ becomes the faster the aggregation occurs, even though the binding strength ⟨*U*_net_⟩ is conceptually different from the intermolecular energy barrier between the bound (aggregate) and unbound states, as mentioned above. Figure 10b suggests, again, that ⟨*U*_net_⟩ and the energy barrier controlling AR are intimately related. As in Figure 9b, the negative portion of ⟨*U*_net_⟩ has been excised from Figure 10a and the absolute value of this portion shown to compare it with the AR in Figure 10b.

An issue for further exploration is the behavior that homopolymeric RNAs (poly­(A), poly­(C), poly­(G), and poly­(U)) would exhibit under Gd and NaCl dialysis. This would eliminate H-bonds, as this is the mechanism for complementary base-pairing, which cannot occur in homopolymeric RNA. The conjecture is that any of the homopolymers of RNA that have π–π stacking would both show the Gd aggregation window and monotonically increase aggregation with [NaCl]. Poly­(A) can form helical structures due to π–π stacking^61^ and poly­(G) can form quadruplexes, so these might display these dialysis behaviors. In contrast, poly­(U) generally does not have secondary structure and poly­(C) only under restrictive solution conditions (acidic), so would be unlikely to have these dialysis behaviors.^62^

### Arrhenius Behavior for Autohydrolysis and Dedimerization Rates

Figure 11 shows that rates for autohydrolysis and dedimerization follow Arrhenius behavior:

$$\mathrm{Rate}=A\;\exp(-\Delta E/k_{B}T)$$ 15

where Δ*E* is the activation energy and *A* is a constant prefactor. In Figure 11, the mRNA was in buffer at pH 5.3, with no added NaCl or Gd. The dissociation rates of dimers and other low-level association are much faster than the autohydrolysis rates, nearly 3 orders of magnitude faster in the case of mRNA #10 and 2 orders of magnitude for mRNA #5. The activation energies for hydrolysis and dedimerization are within each other’s error bars, globally ⟨Δ*E*⟩ = 19.0 kcal ± 4.3 kcal. This is low compared to most protein activation energies for unfolding and aggregation, which are typically 40–150 kcal/mol,^63^ but within the range reported for RNA autohydrolysis.^64,65^ In Figure 11, it is not certain why the activation energies of the two different processes are so similar considering that hydrolysis involves breaking covalent bonds, whereas dedimerization involves physical separation of two intact, associated strands. According to the notion that the secondary structure at least partially controls both aggregations and autohydrolysis, the similarity of the activation energies of the two processes may actually be related to energies required to destroy the secondary structure.

**Figure 11:**
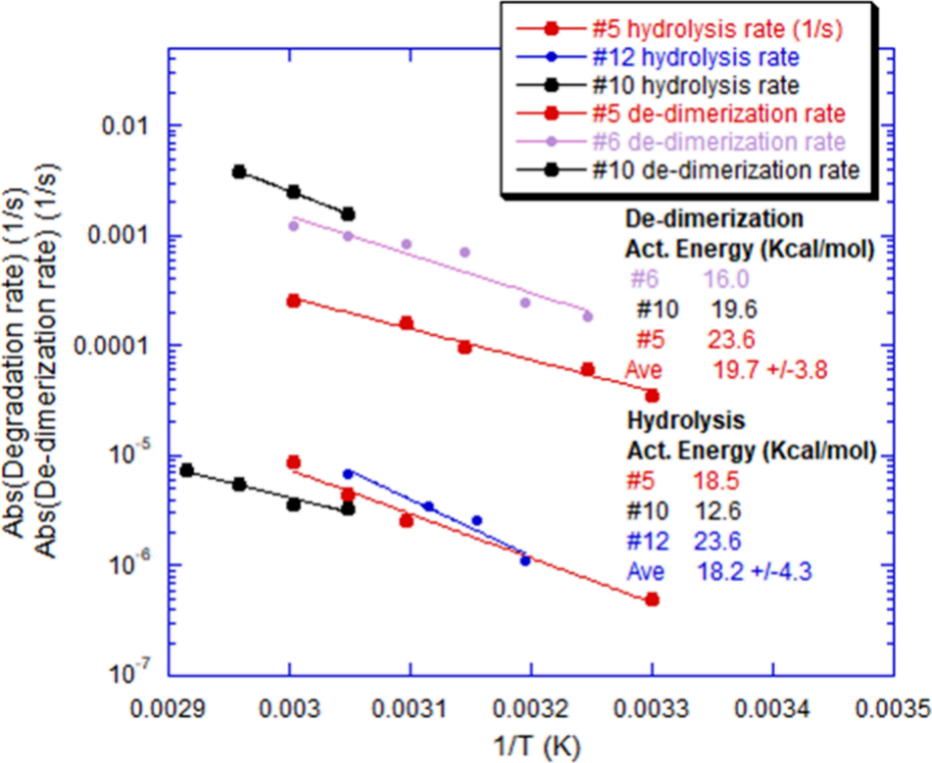
Arrhenius plots for both autohydrolysis rates and dedimerization rates.

### Inclusion of Temperature in the E/HP Model

The E/HP model can explain the abrupt decrease in aggregation rates seen in Figure 5 at the critical temperatures, *T*_c_, by including temperature dependence. A decrease in HP energy with *T* will make ⟨*U*_net_⟩ = 0 in eq 14a at a critical temperature in the aggregation window, *T*_c_. Since ⟨*U*_net_⟩ is negative in the aggregation window, the decrease of the HP effect will lead to the condition ⟨*U*_net_⟩ = 0. In contrast, the electrostatic interactions are only weakly dependent on *T*, as seen in eq 8. Furthermore, since the drop in AR occurs abruptly, it is safe to assume that there is no temperature effect on ⟨*U*_E_⟩ over this narrow range.

According to the last two paragraphs, the *T*-dependence incorporates into the E/HP model as

$$\left\langle U_{\mathrm{net}}\rangle ([\mathrm{Gd}],T)=\langle U_{E}\rangle ([\mathrm{Gd}])+\langle U_{\mathrm{HP}}\rangle ([\mathrm{Gd}],T\right)$$ 16

where ⟨*U*_HP_⟩([Gd], *T*) now shows the explicit dependence on *T*.

Now, it is not critical what form the temperature dependence of ⟨*U*_HP_⟩ takes, as long as it decreases monotonically with increasing *T*. Hence, a convenient form is the Boltzmann-type exponential:

$$\langle U_{\mathrm{HP}}\rangle (T)=Ce^{\Delta E/k_{B}T}$$ 17

which decreases with *T*. Here, *C* is a constant that ensures

$$\langle U_{\mathrm{HP}}\rangle (T_{0})=\langle U_{\mathrm{HP}}\rangle_{0}$$ 18

where *T*_0_ is the reference temperature at which the aggregation window was determined. In this work, *T*_0_ = 25 °C = 298 K. Equation 18 is met for *C* given by

$$C=e^{-\Delta E/k_{B}T_{0}}$$ 19

The temperature-dependent form of eq 11a then becomes

$$\left\langle U_{\mathrm{HP}}\rangle ([\mathrm{Gd}],T)=e^{-\Delta E/k_{B}T_{0}}e^{\Delta E/k_{B}T}[B-\frac{\langle U_{\mathrm{HP}}\rangle_{0}}{1+e^{-\gamma ([\mathrm{Gd}]-[\mathrm{Gd}{]}_{1/2})}}\right]$$ 20

where *B* is given by eq 12. Equation 20 can be more conveniently expressed as

$$\left\langle U_{\mathrm{HP}}\rangle ([\mathrm{Gd}],T)=\exp[\frac{\Delta E}{k_{B}T_{0}}(\frac{T_{0}-T}{T})][B-\frac{\langle U_{\mathrm{HP}}\rangle_{0}}{1+e^{-\gamma ([\mathrm{Gd}]-[\mathrm{Gd}{]}_{1/2})}}\right]$$ 21

This term is negative and dominates over ⟨*U*_E_⟩ in the aggregation window. ⟨*U*_net_⟩ is now given by

$$\left\langle U_{\mathrm{net}}\rangle ([\mathrm{Gd}],T)=\langle U_{E}\rangle ([\mathrm{Gd}])+\exp[\frac{\Delta E}{k_{B}T_{0}}(\frac{T_{0}-T}{T})][B-\frac{\langle U_{\mathrm{HP}}\rangle_{0}}{1+e^{-\gamma ([\mathrm{Gd}]-[\mathrm{Gd}{]}_{1/2})}}\right]$$ 22

The criterion for the phase transition is that ⟨*U*_net_⟩ = 0, which occurs at the experimentally measured *T*_c_. This is illustrated in the inset of Figure 5. Δ*E* can hence be found (or the parameter defining any other type of ⟨*U*_HP_⟩ monotonically increasing with *T* from the corresponding expression for ⟨*U*_net_⟩) from

$$\left\langle U_{E}\rangle ([\mathrm{Gd}])=-\exp[\frac{\Delta E}{k_{B}T_{0}}(\frac{T_{0}-T_{c}}{T_{c}})][B-\frac{\langle U_{\mathrm{HP}}\rangle_{0}}{1+e^{-\gamma ([\mathrm{Gd}]-[\mathrm{Gd}{]}_{1/2})}}\right]$$ 23

or, using eq 11a,

$$\Delta E=\frac{k_{B}T_{c}T_{0}}{\left(T_{0}-T_{c}\right)}\;\ln\left[\frac{\left\langle U_{E}\right\rangle \left(\left[\mathrm{Gd}\right]\right)}{\left|\left\langle U_{\mathrm{HP}}\right\rangle \left(\left[\mathrm{Gd}\right],T_{0}\right)\right|}\right]$$ 24

For this experimental work, *T*_0_ = 25 °C = 298 K; for mRNA #5, *T*_c,5_ = 40 °C = 313 K; and for mRNA #1, *T*_c,1_ = 44 °C = 317 K. Using the parameters from Figure 9a gives ⟨*U*_E_⟩([Gd] = 2 M) = 0.261 eV and ⟨*U*_HP_⟩([Gd] = 2 M, *T*_0_ = 25 °C) = −0.310 eV. This yields

ΔE=0.073eVformRNA#1 25a

ΔE=0.092eVformRNA#5 25b

Note that at *T*_0_ = 298 °C, *k*_B_ *T*_0_ = 0.0257 eV, so that the ratio of Δ*E* to room temperature *kT*_0_ is

$$\frac{\Delta E}{k_{B}T_{0}}=2.84\;\mathrm{for}\;\mathrm{mRNA}\;\#1$$ 26a

$$\frac{\Delta E}{k_{B}T_{0}}=3.59\;\mathrm{for}\;\mathrm{mRNA}\;\#5$$ 26b

The inset of Figure 5 shows ⟨*U*_net_⟩([Gd], *T*) in arbitrary units, according to eq 23 at [Gd] = 2 M, showing the ⟨*U*_net_⟩ = 0 at the experimentally determined *T*_c_ = 40 °C for mRNA #5. It is important to note that while the energy scale in the inset of Figure 7 is in arbitrary units, the ratio $\left[\frac{\left\langle U_{E}\rangle ([\mathrm{Gd}]\right)}{\left|\langle U_{\mathrm{HP}}\rangle ([\mathrm{Gd}],T_{0})\right|}\right]$ in eq 24 should cancel the energy scaling factor for both E and HP, so that the above approximation for Δ*E* should be reasonable.

This finding is interesting in that it indicates that the HP effect is based primarily on π–π stacking (and to a lesser extent on H-bonds), which decreases with *T*, and is the origin of this phenomenon and the existence of *T*_c_, as well as the aggregation itself. It is not the classical hydrophobic effect here, which is operative in irreversible protein aggregation and strengthens as *T* increases.

### Incorporation of a Dimeric Energy Term in the E/HP Model for Interpreting the Rapid Dissociation and Reassociation of mRNA during Up and Down Temperature Ramps

Figure 6a–c, and several figures in Supporting Information all show a rapid decrease in *M*_w_/*M*_0_ at a certain transition temperature *T*_t_. At *T*_t_, *M*_w_/*M*_0_ falls to about one-half its starting value at *T* = 25 °C. This corresponds to a dedimerization or the dissociation of the weight-average of several low levels of dissociation: dimers, trimers, etc. For concreteness, the process will be considered “dedimerization”, while realizing other low-level associations may be involved.

A phenomenological model for the dedimerization focuses on an average net dimerization energy, ⟨*U*_net,d_⟩. This energy is different in origin than the sum of intermolecular electrostatic and HP energies in the intermolecular E/HP model of eqs 7–14a and 16–24, which lead to massive, reversible, or semireversible aggregation. In the case of these massive aggregates, electrostatic energy between mRNA molecules decreases as IS increases, allowing H-bonding between base pairs and π–π stacking to cause extended aggregation of a dozen or more mRNA. These aggregations may be qualitatively different than dimers, highly random, less stable, and requiring less energy to dissociate than the dimers, which may have more specific base-pair associations.

Due to the fact that *T*_t_ decreases with [NaCl], as seen in Figure 7, the temperature-dependent dedimerization energy, ⟨*U*_net,d_⟩ must now be a function of [NaCl] and *T*, ⟨*U*_net,d_⟩([NaCl], *T*). There may be many possible dimerization combinations of base-pairing, each with its own dimerization energy, so that there is a variety of dimerization states in the mRNA population. There have been reports of such varied dimerization states.^1^ This may explain why the decrease in *M*_w_/*M*_0_ begins at a low temperature, around 33 °C in Figure 6a; some dimerization states are less stable than others and so dedimerize at lower *T*. This may also explain some of the hysteresis in Figure 6a; when dissociated and then reassociated, only a subset of the original dimerizations may reoccur.

The net dimerization energy ⟨*U*_net,d_⟩([IS], *T*) is composed of an intradimer electrostatic repulsion, which decreases monotonically with [IS] and is essentially *T*-independent, ⟨*U*_E,d_⟩([*IS*]), and an attractive intradimer energy term which subsumes the various dimer stabilizing effects, ⟨*U*_S,d_⟩([IS], *T*). The subscript “S”, for “stabilization”, indicates a combination of base-pairing, π–π, and other possible hydrophobic effects but different in their magnitudes and mechanisms than the ‘HP’ effect subscript used in the intermolecular E/HP model. Hence, the term ⟨*U*_S,d_⟩([IS], *T*) is more complex than ⟨*U*_E,d_⟩([IS]), as it involves the several effects mentioned.

Empirically, there is a measurable *T*_t_([IS]) around which the rapid dedimerization occurs, seen in Figure 7. Because HP effects contribute cooperatively to the stabilization energy, ⟨*U*_S,d_⟩([IS], *T*) can be modeled by a sigmoid, as is done in modeling many cooperative phenomena, taken to be of the same form as the logistic equation in eq 11a, but involving different parameters:

$$\langle U_{S,d}\rangle ([\mathrm{IS}],T)=-\frac{\left\langle U_{S,d}\rangle_{0}([\mathrm{IS}]\right)}{1+e^{\gamma_{d}(T-T_{t}([\mathrm{IS}]))}}$$ 27

where γ_d_ is positive and controls the width of the sigmoid and ⟨*U*_S,d_⟩_0_([IS]) is the absolute magnitude of the HP stabilization energy at no added IS (the negative sign in eq 27 makes ⟨*U*_S,d_⟩([IS], *T*) negative, and it is a decreasing function of [IS], although not necessarily monotonic, given the “bump” that appears before *T*_t_ in data for all the mRNA tested). The net dimeric energy is then

$$\langle U_{\mathrm{net},d}\rangle ([\mathrm{IS}],T)=\langle U_{E,d}\rangle (\mathrm{IS})-\frac{\left\langle U_{S,d}\rangle (\mathrm{IS}\right)}{1+e^{\gamma_{d}(T-T_{t}([\mathrm{IS}]))}}$$ 28

At *T*_t_, ⟨*U*_net,d_⟩([IS], *T*_t_) = 0, which is the temperature of dedimerization on the up-ramp. This condition implies that ⟨*U*_E,d_⟩(IS) = ⟨*U*_S,d_⟩(IS) in this model, and

$$\left\langle U_{\mathrm{net},d}\rangle ([\mathrm{IS}],T)=\langle U_{S,d}\rangle (\mathrm{IS})[\frac{2}{1+e^{\gamma_{d}(T-T_{t}([\mathrm{IS}]))}}-1\right]$$ 29

This form captures the abrupt transition at *T*_t_, where a fit to sigmoidal eq 27 is shown over the transition region in Figure 6a (green), with the following fit parameters: *T*_t_ = 57.06 °C, γ_d_ = 3.608. However, this model does not provide a form of *T*_t_ (IS). Figure 7 shows a phenomenological power law fit to the experimental *T*_t_ versus [NaCl] and the transition temperature on the down-ramp. The two temperatures are slightly displaced from each other, but both follow the same empirical power law versus [NaCl], with an exponent of 0.12.

### Correlation between Thermal Hydrolysis and Aggregation Rates in Gd

There is a good correlation between the maximum mRNA AR within the aggregation window and the autohydrolysis rate in both acetate and 6 M Gd at *T* = 55 °C after 5 days. The hydrolysis rates, as defined here, do not correlate strongly with the number of base pairs. The maximum AR at *T* = 25 °C occurs around 2 M [Gd]. Figure 12 shows the close covariance between autohydrolysis rates at *T* = 55 °C for each mRNA in both acetate buffer and 2 M [Gd], and AR in 2 M Gd solution at *T* = 25 °C; i.e., the greater the AR, the greater the autohydrolysis rate, suggesting loss of secondary and/or tertiary structure-exposed nucleobases, making them both more vulnerable to autohydrolysis and π–π stacking-based aggregation.

**Figure 12:**
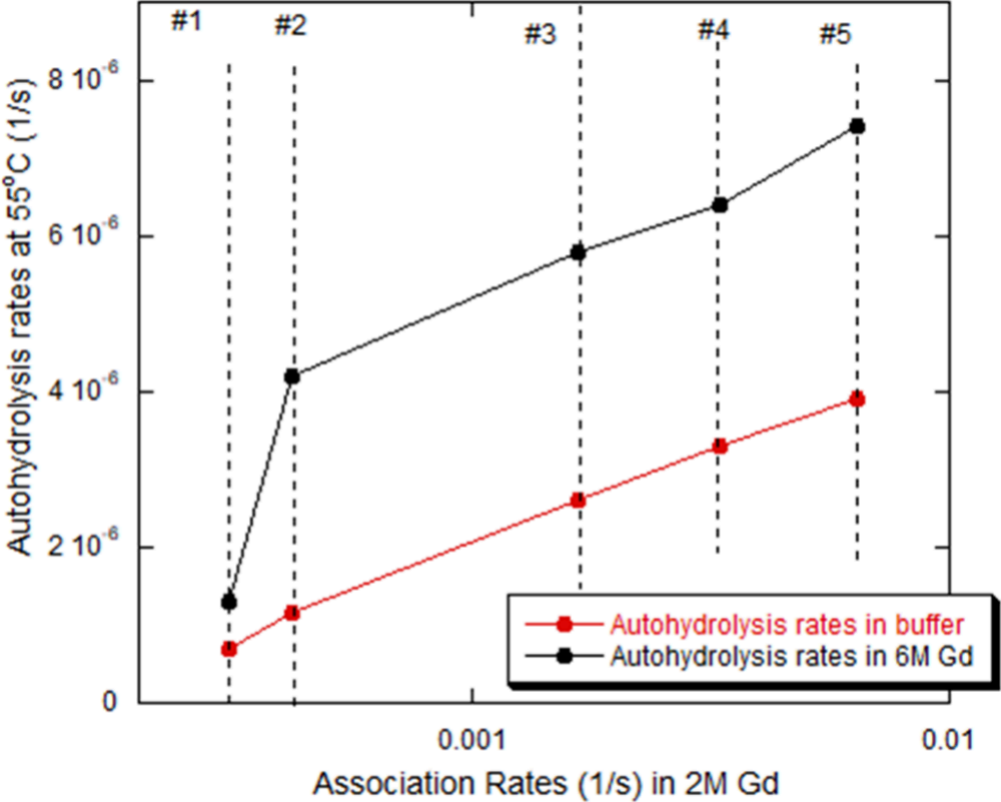
Close correlation between thermal degradation rates and AR for mRNA #1, #2, #3, #4, and #5.

This correlation leads back to the notion of widely varying types of secondary, and possibly tertiary structure, and how these structures seemingly protect mRNA from both thermal degradation via autohydrolysis and aggregation rates in partially denaturing Gd solutions at *T* = 25 °C. The higher the thermal plateaus and the lower the AR, the more “persistent secondary structure” in the mRNA.

## Conclusions

Free mRNA in the solution displays a rich profile of physical solution properties, including (i) reversible and semireversible massive colloidal aggregation; (ii) rapid low-level associations and dissociations of dimers and possibly trimers, tetramers, etc., with ionic strength-dependent transition temperature *T*_t_; (iii) autohydrolysis leading to temperature-dependent final plateaus of *M*_w_, rather than continuing on to complete hydrolysis; and (iv) abrupt, orders of magnitude decrease in aggregation at and above a certain critical temperature *T*_c_. A central hypothesis of this work is that these disparate phenomena are connected via the secondary (and possibly tertiary) structure of mRNA, and this structure exists in several manifestations, involving different numbers of nucleotides, giving each type of mRNA a different physical profile and different levels of robustness against colloidal and low-level associations and autohydrolysis. The secondary structure appears to partially protect against both of these unwanted processes. When secondary structure is lost, it exposes nucleotides to quicker autohydrolysis and more extensive intermolecular associations. The methods here may help to establish robustness scales for mRNA, and if sequence information is available, correlations might be made between robustness, secondary structure, and sequence.

The authors could find no previous publications experimentally demonstrating that free mRNA in solution can massively aggregate due to the presence of simple, monovalent electrolyte (NaCl), and to the presence of a denaturing agent (Gd) in which most reports consider mRNA highly soluble (not aggregated).

The light scattering during dialysis in a spectroscopic cuvette provides a unique method of assessing the effects of electrolytes, surfactants, and other excipients, automatically and over a large concentration range in a single experiment. The dialysis method allows automatic and continuous monitoring of excipient effects over broad concentration ranges, which might be used in lieu of 96 or more discrete concentrations measured in well plates.

The use of a simple electrolyte, NaCl, and the chaotropic (denaturant) agent guanidine-HCl (Gd) allows experiments to be designed that distinguish between purely electrostatic (E) interactions and interactions involving H-bond, π–π stacking, and the classical hydrophobic effect (HP effects). A phenomenological E/HP energy model was developed to account for the colloidal aggregation behavior, including the critical temperature *T*_c_. It explains the “aggregation window” found for mRNA dialyzed against Gd, whereby the ionic strength due to Gd allows HP attractions to form at around 0.5 M Gd and persist up until about 4 M Gd, after which the HP effects causing the aggregates are destroyed by the Gd. In contrast, aggregation increases monotonically with [NaCl], as NaCl does not affect the HP effects.

The model was further extended to dimer and other low-level associations and dissociations consistently seen during temperature up- and down-ramps. Measurements at fixed *T*, fixed NaCl, and fixed Gd gave complementary data on rates of autohydrolysis and aggregation. Arrhenius behavior was found for the aggregation rates (ARs), thus connecting to the energetics of the model, which gives regimes of stability.

Table 2 summarizes the various phenomena, their relationship to Gd and NaCl, and the method that was used for the determination.

**Table 2:** Summary of the Various Phenomena and Their Physical Implications^a^

phenomenon	Gd	NaCl	method	applies to tail-less RNA?	physical implication
association window	0.5–3 M	no	dialysis, [iso-Gd]	yes, but broader window	interplay of electrostatic and hydrophobic effects
AR follows window	Yes	NA*	[iso-Gd]	yes	⟨*U*_net_⟩ and repulsive barrier height are linked
monotonic aggregation increases and follows AR	NA*	0.5–5 M	dialysis, iso-[NaCl]	yes, in NaCl	⟨*U*_net_⟩ and repulsive barrier height are linked
reversible aggregation	yes	partial	dialysis	partial in Gd and NaCl	aggregation largely reversible in NaCl and Gd
hysteresis on down-ramp	no data	>25 mM	*T*-ramp, up and down	no data	complex changes in self-association, *T*-dependent
AR vs *T*, abrupt drop at *T*_c_	2 M	no data	Iso-T	yes	evidence that π–πstacking is principal origin of massive aggregation
dedimerization	no	yes	Iso-T	no data	reversible dimer/low-level associations
dedimerization sharp transition at *T*_t_	no data	>25 mM		no data	fundamental dedimerization or dissociation of trimers, etc. common to all mRNA tested
autohydrolysis kinetics	0, 6 M	0, 1 M	Iso-T		*T*-dependent and slow compared to Self-dissociation kinetics
hydrolysis plateaus final *M*_w_/*M*_0_ values vs T	0, 6 M	0, 1 M	Iso-T	yes	evidence of persistent, *T*-dependent secondary structure
correlated AR and hydrolysis rates	2 M	no data	Iso-T and iso-Gd	no data	secondary structure impedes both hydrolysis and aggregation
E/HP model works	yes	yes	dialysis, Gd, and NaCl		supports competing repulsive electrostatic\forces and attractive HP effects

a NA* = not applicable.

The identification of a guanidine-dependent aggregation window for mRNA, along with the elucidation of the balance between electrostatic repulsion and attractive interactions—namely, hydrogen bonding, π-π stacking, and hydrophobic effects (collectively HP)—has important implications for the development of mRNA therapeutics. These findings demonstrate that mRNA low-level association and aggregation are acutely sensitive to both ionic strength and temperature, offering a mechanistic basis for the rational design of formulation buffers that minimize aggregation during manufacturing, storage, and administration. Notably, the absence of an aggregation window in NaCl-containing solutions versus its emergence in the presence of guanidine hydrochloride (Gd) underscores the risks associated with employing chaotropic or denaturing agents without precise control of the concentration. Furthermore, the observed sharp decline in aggregation rates above ∼40 °C suggests that mRNA molecules may retain structural integrity during transient thermal excursions provided that their secondary structures remain largely intact—an insight that can inform the design of more robust cold-chain and transport strategies. Finally, the strong correlation between aggregation propensity and resistance to autohydrolysis highlights the protective role of a persistent secondary structure. This suggests that engineering mRNA sequences with stable secondary motifs may enhance both their physical durability and their therapeutic efficacy. Collectively, these results support a structure-informed approach to the formulation and storage of mRNA, with the aim of preserving the molecular integrity and ensuring functional delivery in clinical applications.

All of the results in this work regard the physical chemistry of free mRNA in solution. They do not predict how mRNA behaves when inside a lipid nanoparticle or when interacting in the solution with other polymers such as proteins.

## Supplementary Material



## Notes

Extra data for dialysis for more of the mRNA types in Table 1 are given, as well as the results for temperature up and down-ramps for many of the mRNA types, isothermal data for several more mRNA types, time-dependent iso-ionic data using NaCl and Gd on various other mRNA types in Table 1 (PDF)

## Glossary

AR: aggregation rate (1/s)
⟨*D*_0_⟩_*z*_: *z*-averaged self-diffusion coefficient (low concentration)
*D*_H,ap_: apparent hydrodynamic diameter (eq 2b)
HP: H-bonding, π–π stacking, and other hydrophobic effects
IS: ionic strength
Gd: guanidine-HCl
*M*_w_: weight-average molar mass
⟨*S* ^2^⟩_*z*_^1/2^: root-mean-square *z*-average radius of gyration
*T*_c_: critical temperature within the aggregation window where aggregation abruptly slows dramatically
*T*_t_: Transition temperature during a temperature ramp at which there is an abrupt loss of low-level aggregations such as dimers.
