# Supplementary material for: Consequences of mRNA Secondary Structure on Stability against both Hydrolysis and Aggregation: The Role of Electrostatic, π–π Stacking, and Thermal Effects
Source: ACS Omega. 2026 Jan 8;11(3):4440–56. doi: 10.1021/acsomega.5c10266 (PMC12854604; doi:10.1021/acsomega.5c10266)
Supplement: Supplementary file 1 [file ao5c10266_si_001.pdf]

Supporting Material for

Consequences of mRNA Secondary Structure on Stability against both Hydrolysis and Aggregation; The Role of Electrostatic, π–π stacking, and Thermal Effects

Curtis W. Jarand,[1] Zhiyou Deng,[2] Mark L. Brader,[2] Wayne F. Reed[*,1]

[1]Tulane University, New Orleans, Louisiana, 70118, USA

[2]Moderna, Inc., Cambridge, Massachusetts, USA

[*]Correspondence. wreed@tulane.edu

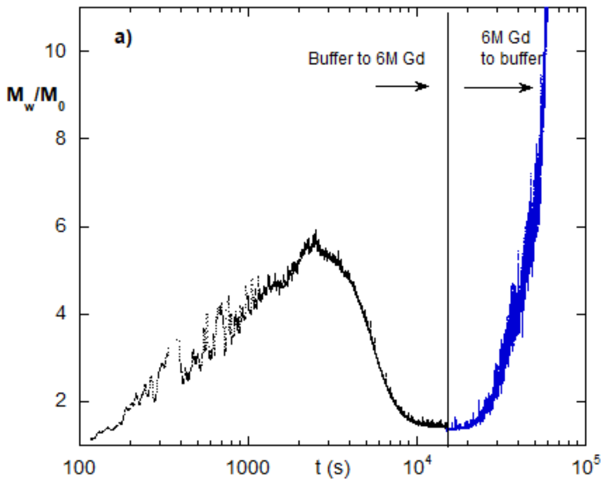

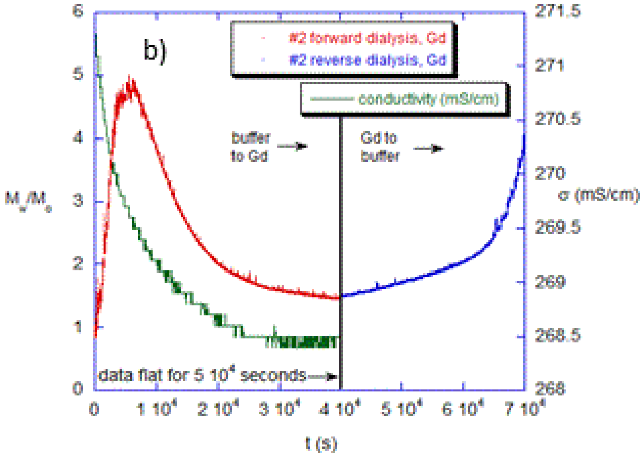

b) 1

Figure S1a. Forward dialysis (sample starting in buffer dialyzed against 6M Gd) and reverse dialysis (dialysis against buffer following forward dialysis) of mRNA #1

Figure S1b. Forward dialysis (sample starting in buffer dialyzed against 6M Gd) and reverse dialysis (dialysis against buffer following forward dialysis) of mRNA #2

The end of the forward dialysis and beginning of the reverse dialysis is truncated to more clearly show the changes in M_w/M_o during dialysis. The conductivity of the dialysate is shown for the forward dialysis. The Gd Association Window is apparent, and is the peaked red curve.

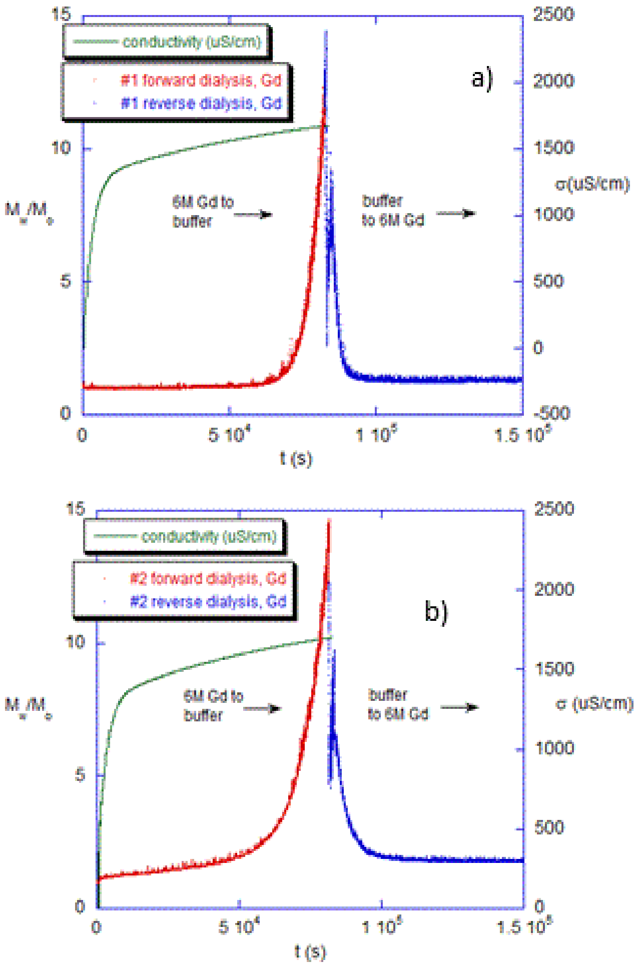

Figure S2a. Forward dialysis (sample starting in 6M Gd dialyzed against buffer) and reverse dialysis (dialysis against 6M Gd following forward dialysis) of mRNA #1. The conductivity of the dialysate is shown for the forward dialysis.

Figure S2b. Forward dialysis (sample starting in 6M Gd dialyzed against buffer) and reverse dialysis (dialysis against 6M Gd following forward dialysis) of mRNA #2. The conductivity of the dialysate is shown for the forward dialysis.

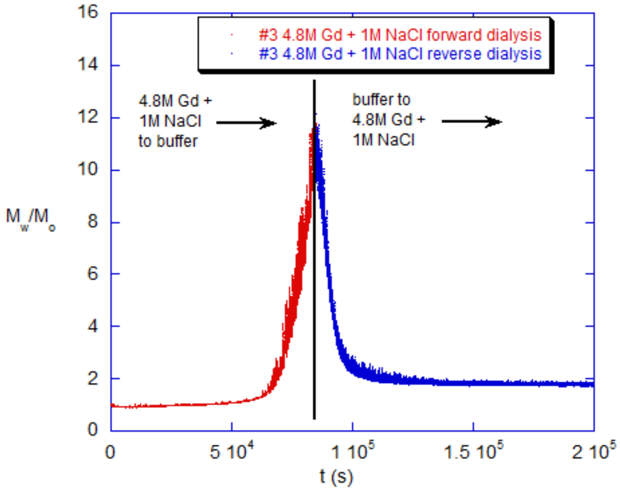

Figure S3. Forward dialysis (sample starting in 4.8M Gd and 1M NaCl dialyzed against buffer) and reverse dialysis (dialysis against 6M Gd following forward dialysis) of mRNA #3.

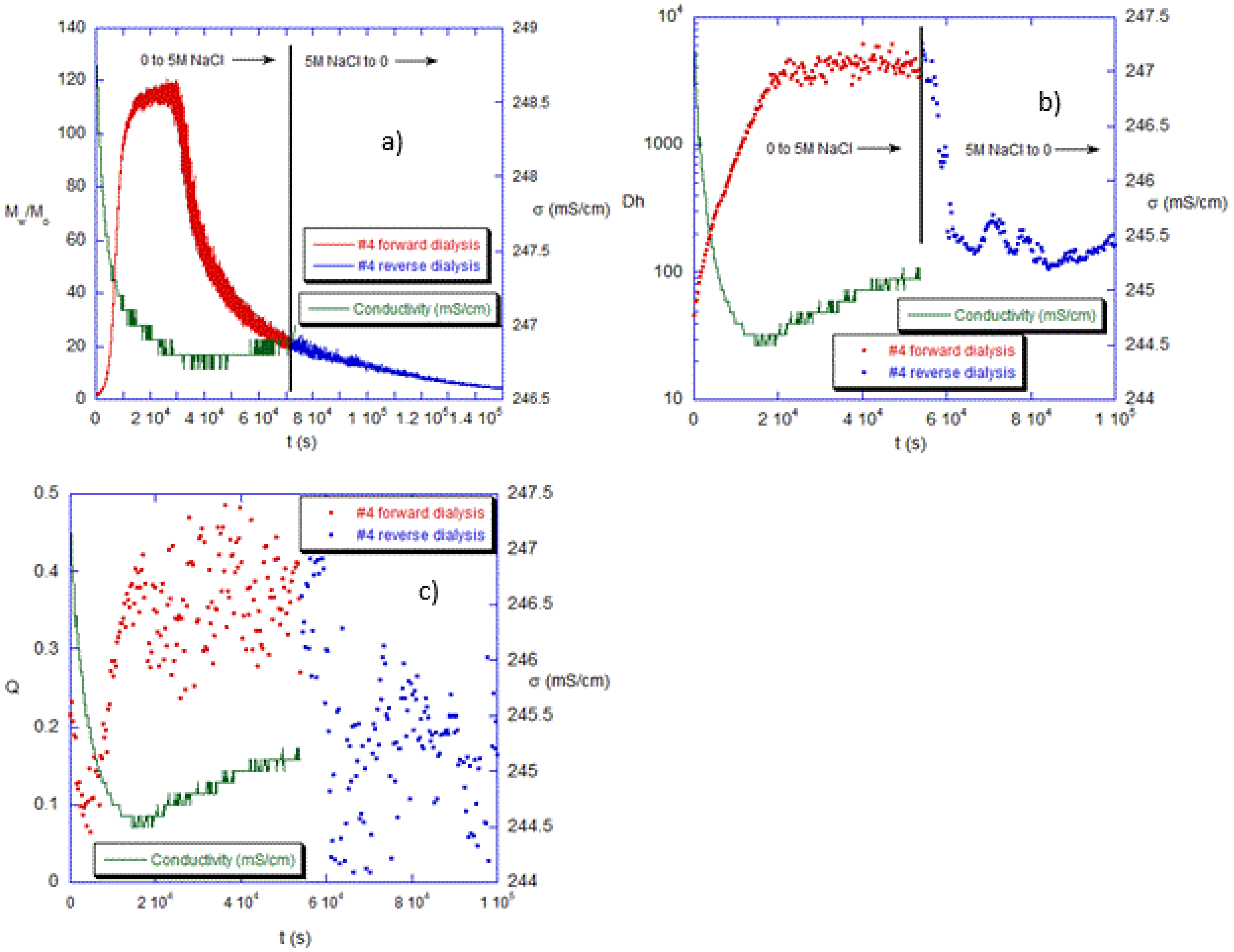

Figure S4a. M_w/M_0 data for the forward and reverse dialysis of mRNA #4 starting in Ac buffer dialyzed against 5M NaCl (forward) then dialyzed back against Ac buffer (reverse). There is precipitation occurring in the forward dialysis, seen both by the drop in M_w/M_0.

Figure S4b. Dh data for the forward and reverse dialysis of mRNA #4 starting in Ac buffer dialyzed against 5M NaCl (forward) then dialyzed back against Ac buffer (reverse). There is precipitation occurring in the forward dialysis, seen both by the drop in Dh above the practical limit of DLS measurements (~3μm).

Figure S4c. Polydispersity, Q, data for the forward and reverse dialysis of mRNA #4 starting in Ac buffer dialyzed against 5M NaCl (forward) then dialyzed back against Ac buffer (reverse). There is precipitation occurring in the forward dialysis, seen by the very large values of Q (>0.25, considered highly polydisperse).

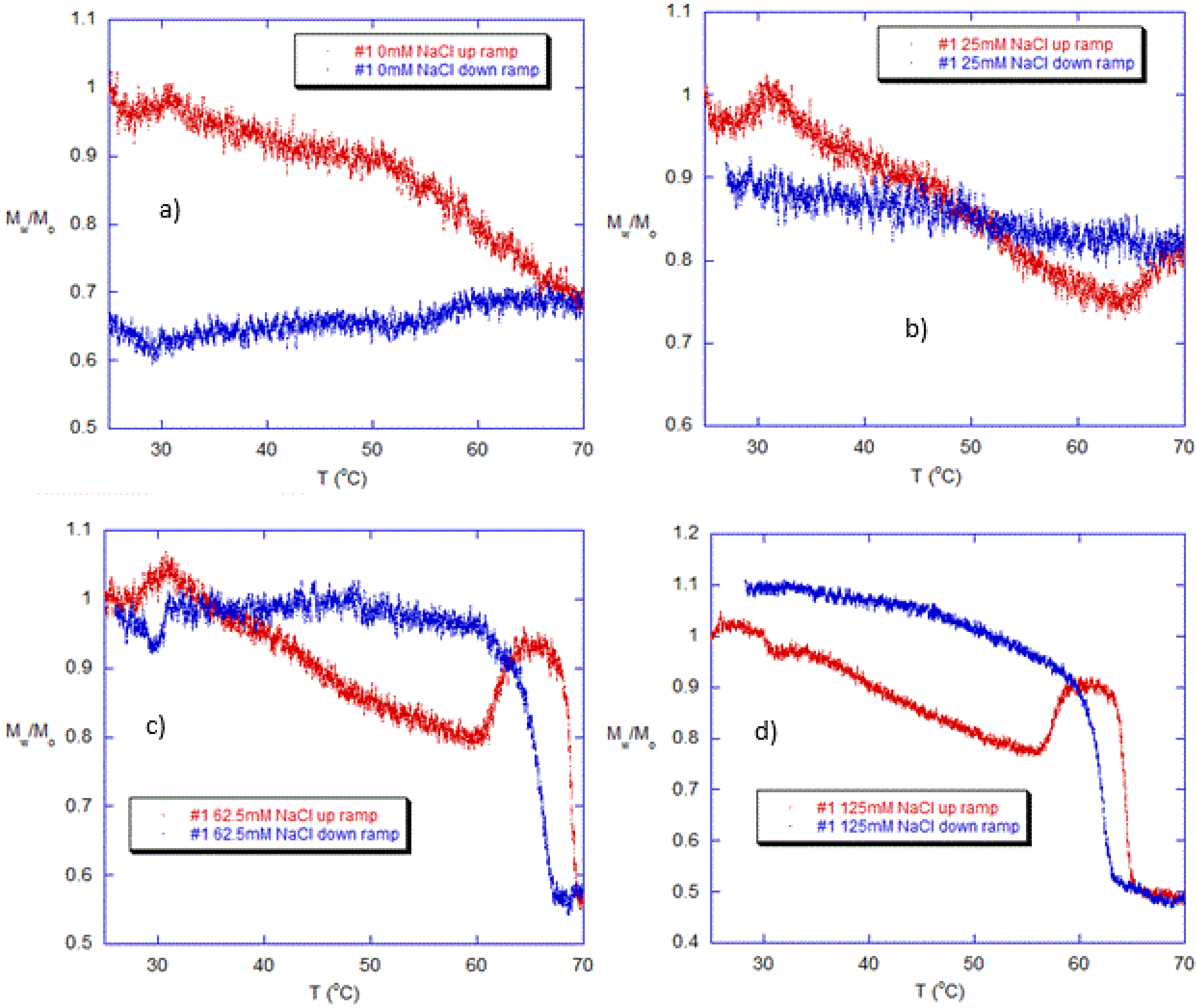

a)
#1 0mM NaCl up ramp
#1 0mM NaCl down ramp
b)
#1 25mM NaCl up ramp
#1 25mM NaCl down ramp
c)
#1 62.5mM NaCl up ramp
#1 62.5mM NaCl down ramp
d)
#1 125mM NaCl up ramp
#1 125mM NaCl down ramp
M_w/M_o
T (^oC)

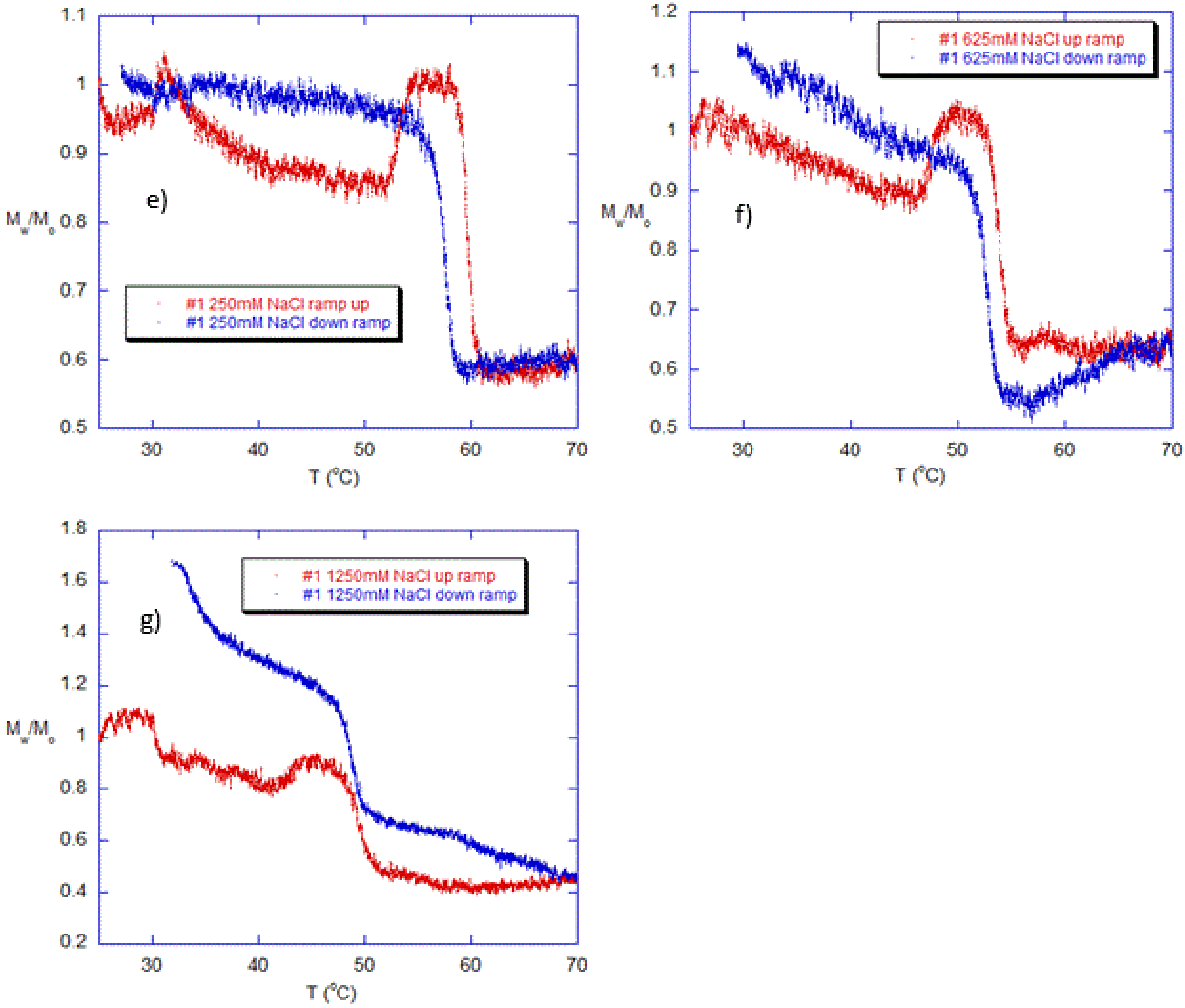

Figure S5a. Temperature ramps from 25°C to 70°C (up) and 70°C to <30°C (down) for mRNA #1 in varying NaCl. Ramp rates were ±0.25°C/minute. 0mM NaCl.

Figure S5b. 25mM NaCl

Figure S5c. 62.5 mM NaCl

Figure S5d. 125 mM NaCl

Figure S5e. 250mM NaCl

Figure S5f. 625 mM NaCl

Figure S5g. 1250 mM NaCl

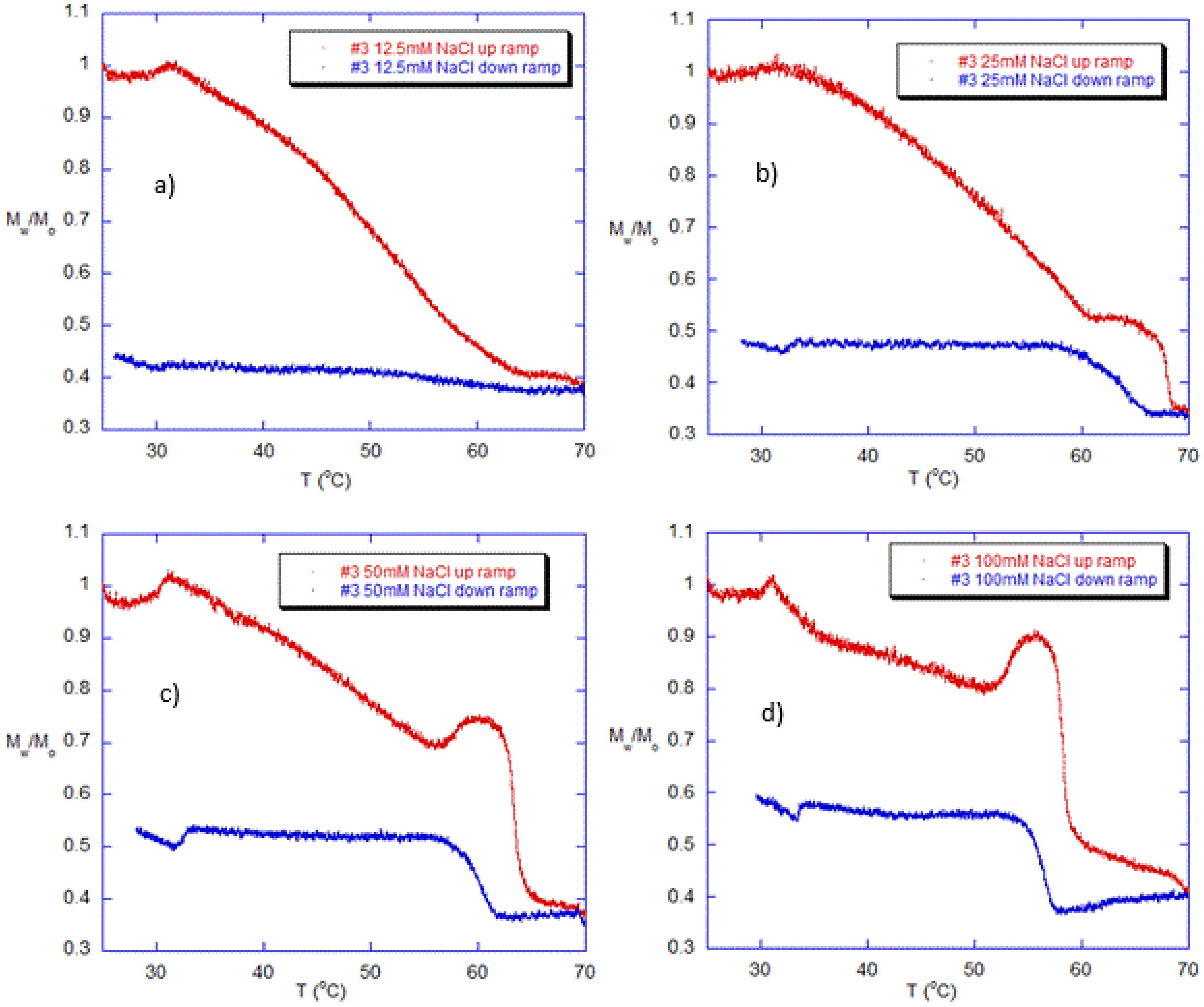

Figure S6a-d. Temperature ramps from 25°C to 70°C (up) and 70°C to <30°C (down) for mRNA #3 with varying concentrations of NaCl. Ramp rates were ±0.25°C/minute. 12.5mM NaCl.

Figure S6b. 25mM NaCl.

Figure S6c. 50 mM NaCl.

Figure S6d. 100 mM NaCl.

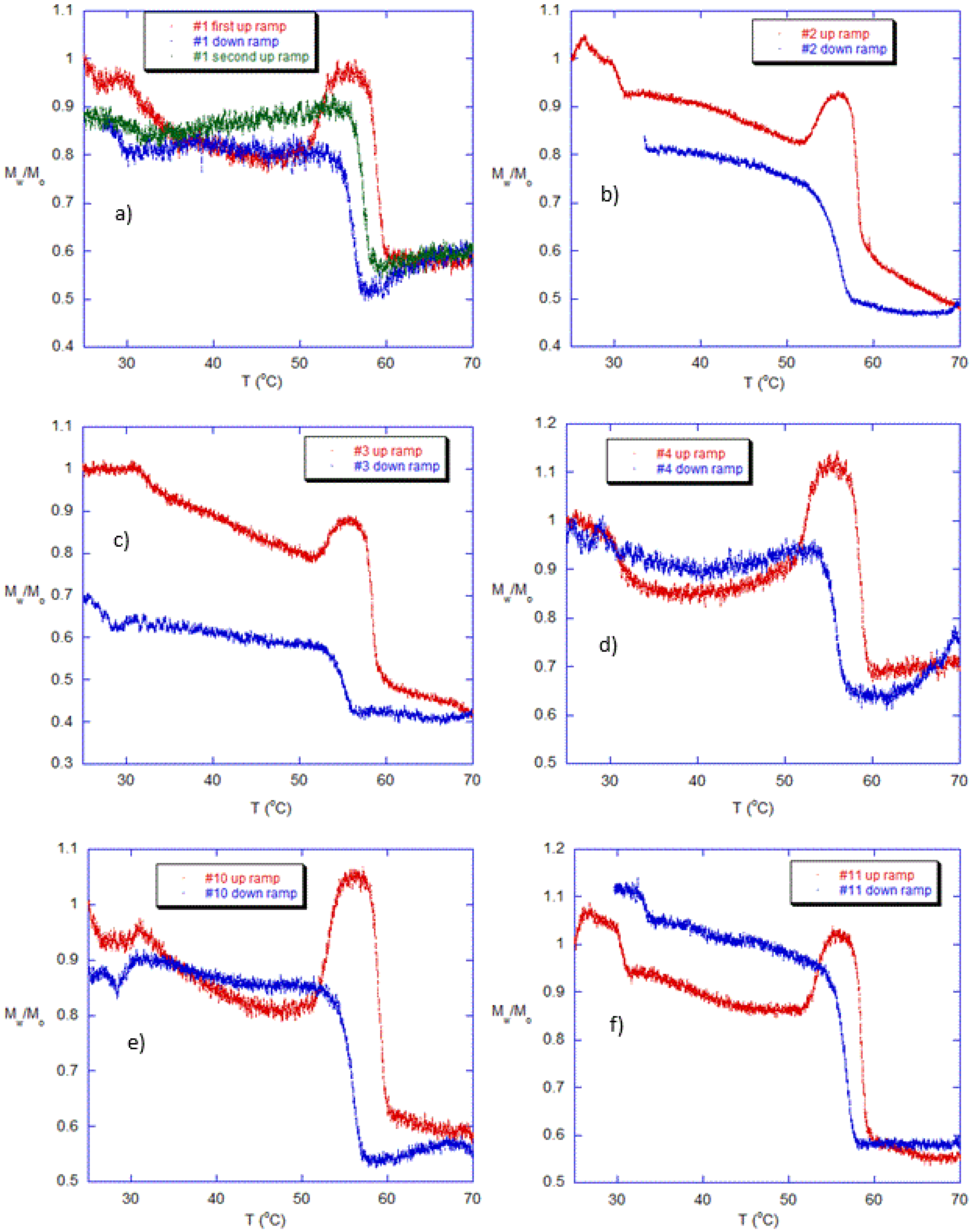

a)
#1 first up ramp
#1 down ramp
#1 second up ramp
b)
#2 up ramp
#2 down ramp
c)
#3 up ramp
#3 down ramp
d)
#4 up ramp
#4 down ramp
e)
#10 up ramp
#10 down ramp
f)
#11 up ramp
#11 down ramp
M_w/M_o
T (°C)

Figure S7a. Temperature ramps from 25°C to 70°C (up) and 70°C to <30°C (down) for mRNAs 1-4, 10 and 11 in 100mM NaCl. Ramp rates were ±0.25°C/minute. A second up ramp was conducted on mRNA 1 and shows the loss of the hump that occurs in all tested mRNAs between 50°C to 60°C. mRNA #1

Figure S7b. mRNA #2

Figure S7c. mRNA #3

Figure S7d. mRNA #4

Figure S7e. mRNA #10

Figure S7f. mRNA #11

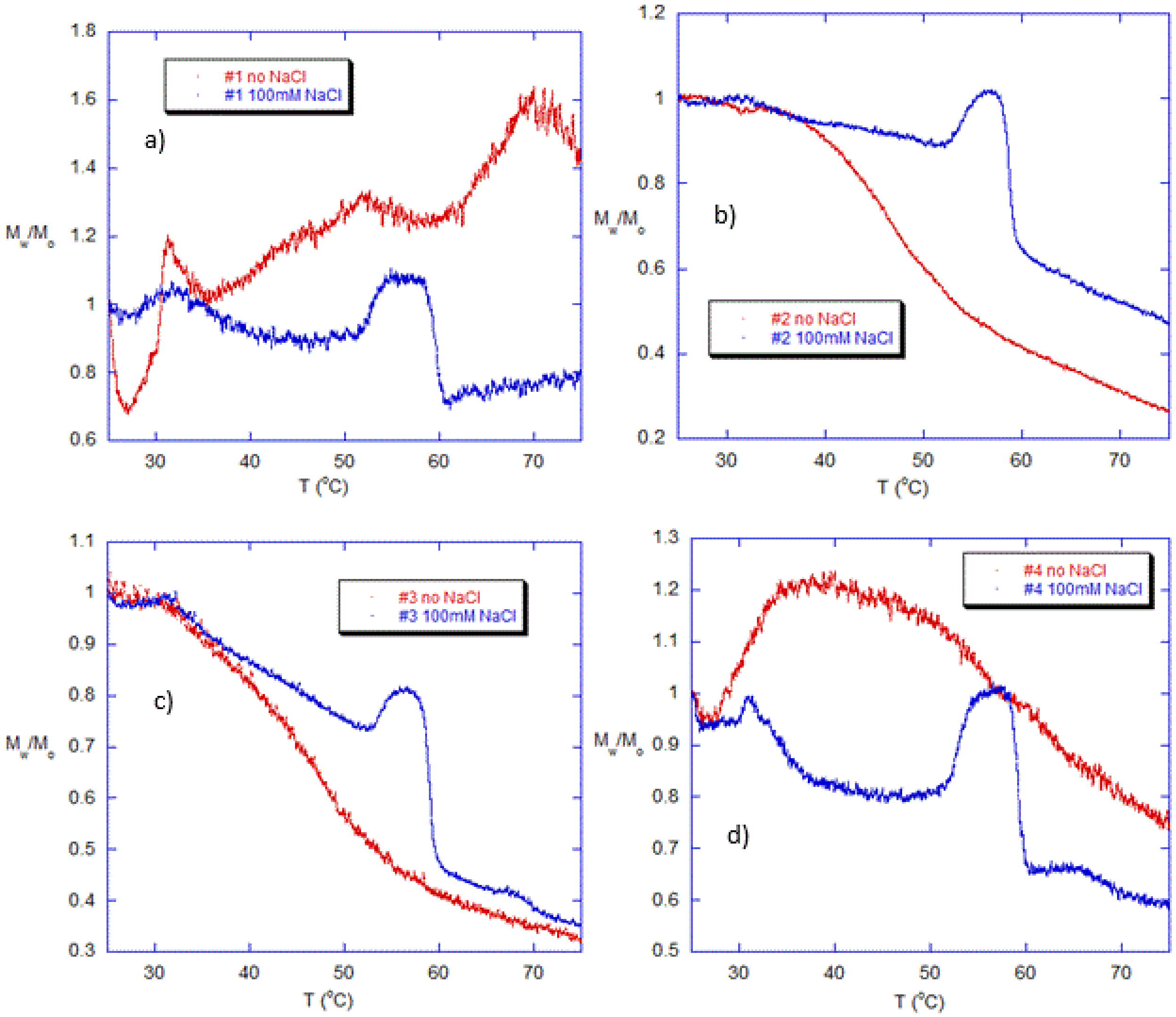

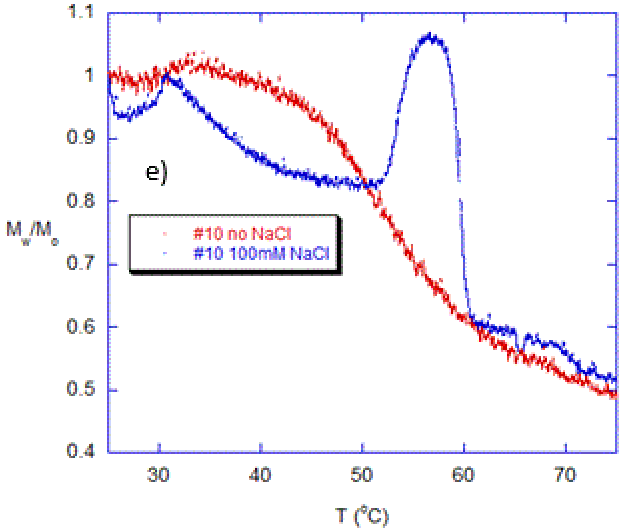

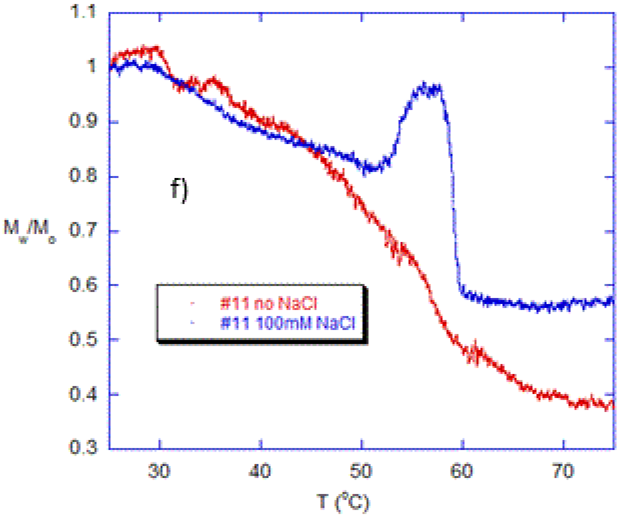

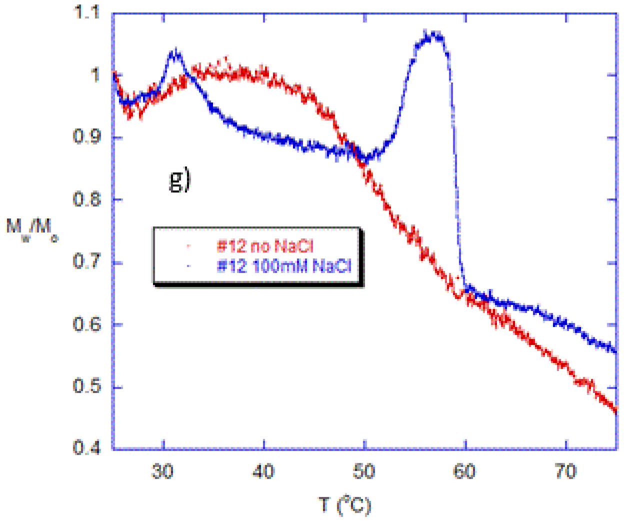

Figure S8a. Temperature ramps from 25°C to 75°C at a ramp rate of 0.277°C/min for mRNAs 1-4 and 10-12 in 0mM and 100mM NaCl.

Figures S8b. mRNA #2.

Figures S8c. mRNA #3.

Figures S8d. mRNA #4.

Figures S8e. mRNA #10.

Figures S8f. mRNA #11.

Figures S8g. mRNA #12.

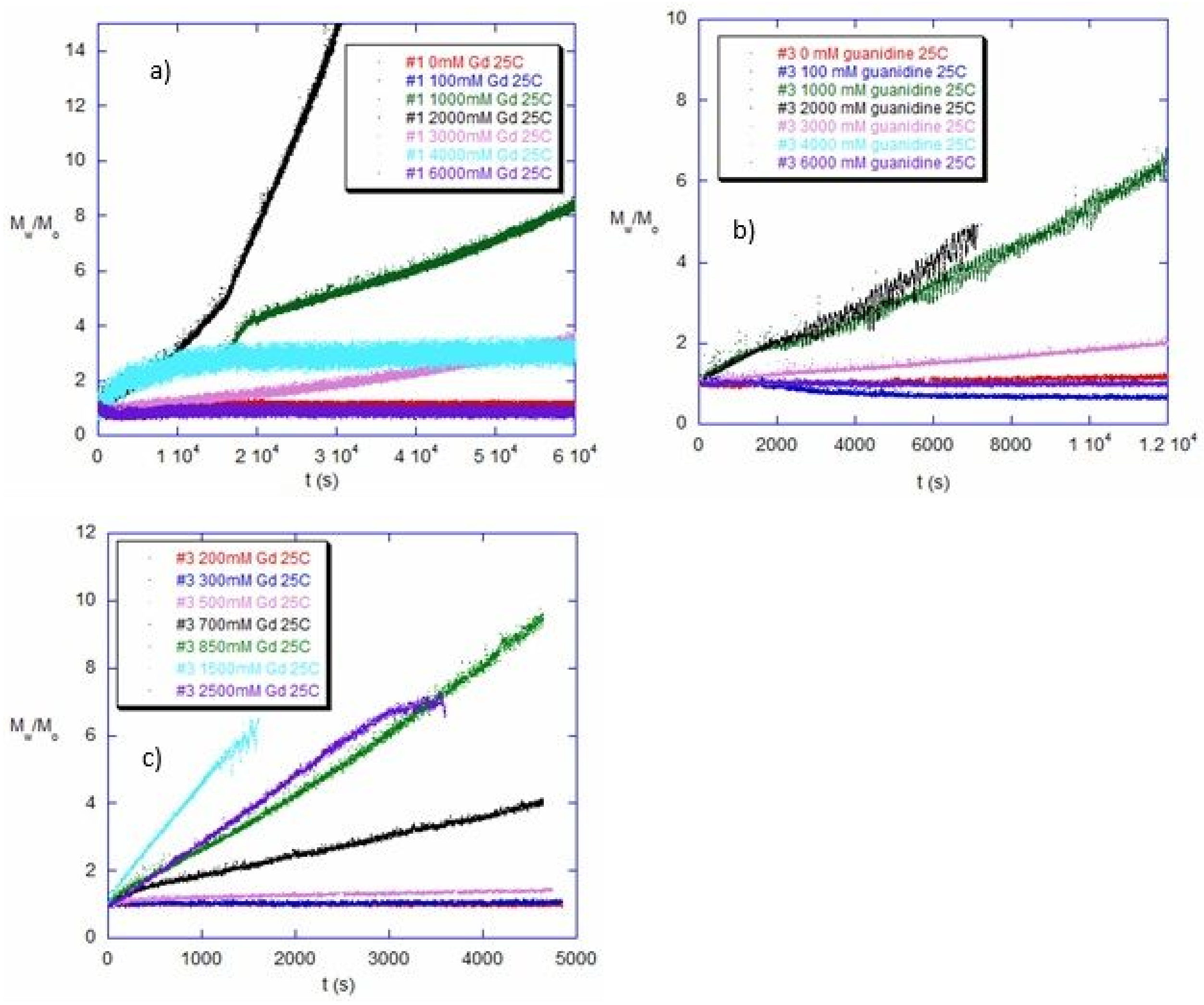

Figure S9a. mRNA #1 in varying concentrations of Gd with a 25°C isothermal hold.

Figure S9b. mRNA #3 in varying concentrations of Gd with a 25°C isothermal hold.

Figure S9c. mRNA #3 in concentrations of Gd complementary to Figure s9b.

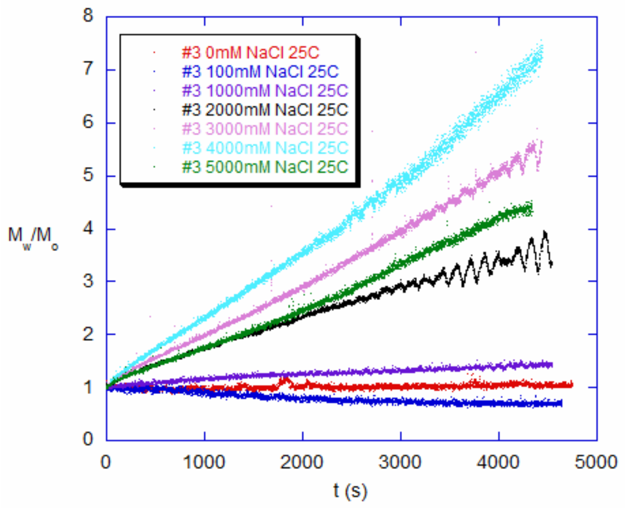

Figure S10. mRNA #3 in varying concentrations of NaCl with a 25°C isothermal hold.

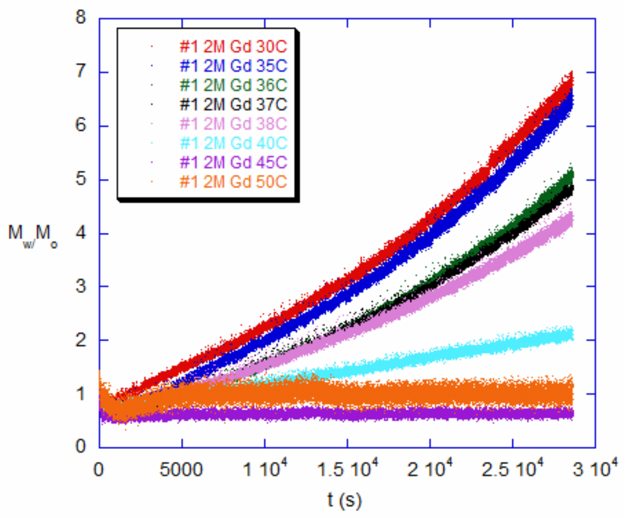

Figure S11. mRNA #1 in 2M Gd with isothermal holds ranging from 30-50°C.

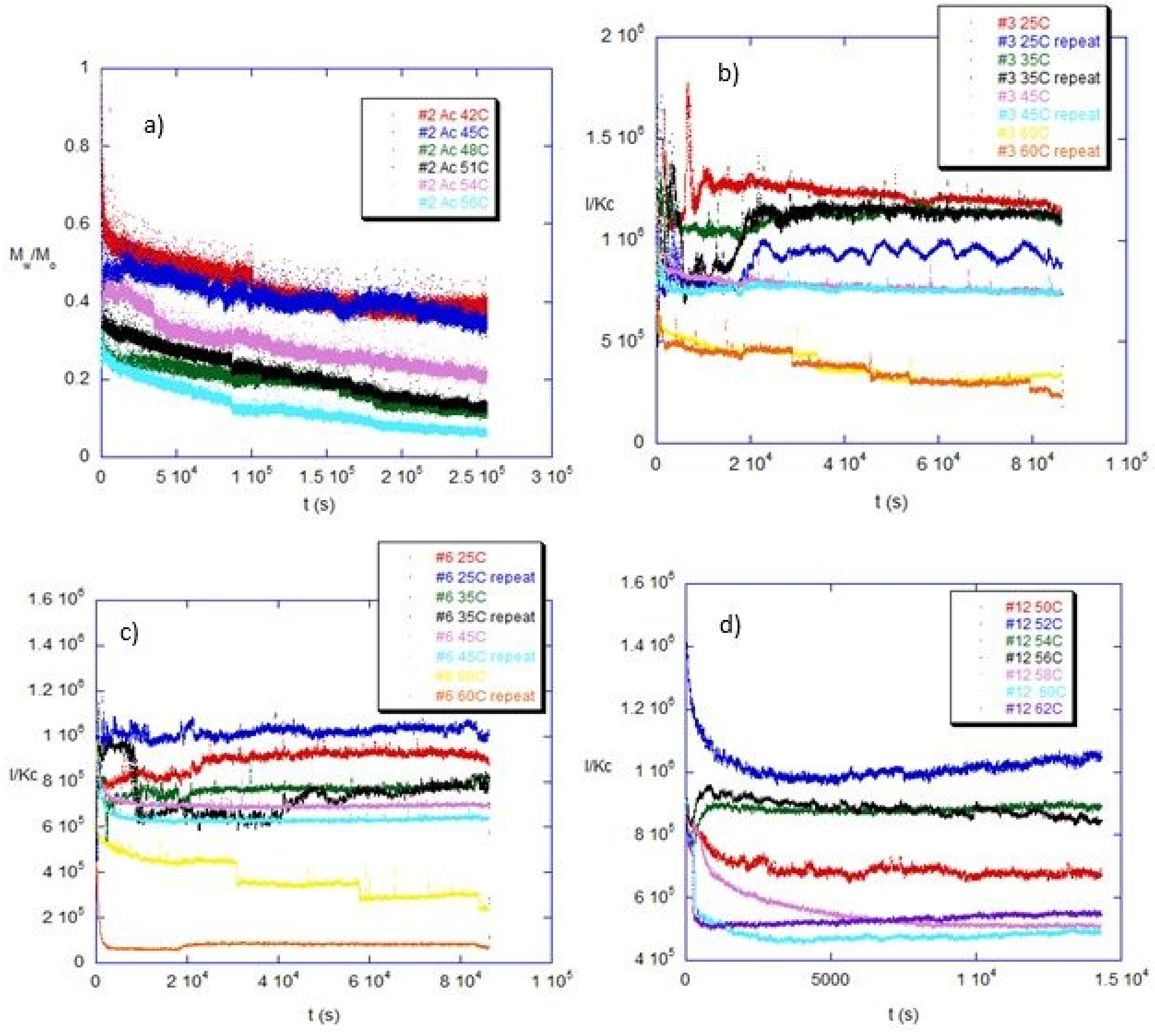

.

Figure S12a. mRNA #2,3,6 and12 in Ac buffer with isothermal holds between 25-62°C.

Figure S12b. mRNA #3

Figure S12c. mRNA #6

Figure S12d. mRNA #12
